# Optimizing genomic reference populations to improve crossbred performance

**DOI:** 10.1186/s12711-020-00573-3

**Published:** 2020-11-06

**Authors:** Yvonne C. J. Wientjes, Piter Bijma, Mario P. L. Calus

**Affiliations:** Wageningen University & Research, Animal Breeding and Genomics, 6700 AH Wageningen, The Netherlands

## Abstract

**Background:**

In pig and poultry breeding, the objective is to improve the performance of crossbred production animals, while selection takes place in the purebred parent lines. One way to achieve this is to use genomic prediction with a crossbred reference population. A crossbred reference population benefits from expressing the breeding goal trait but suffers from a lower genetic relatedness with the purebred selection candidates than a purebred reference population. Our aim was to investigate the benefit of using a crossbred reference population for genomic prediction of crossbred performance for: (1) different levels of relatedness between the crossbred reference population and purebred selection candidates, (2) different levels of the purebred-crossbred correlation, and (3) different reference population sizes. We simulated a crossbred breeding program with 0, 1 or 2 multiplication steps to generate the crossbreds, and compared the accuracy of genomic prediction of crossbred performance in one generation using either a purebred or a crossbred reference population. For each scenario, we investigated the empirical accuracy based on simulation and the predicted accuracy based on the estimated effective number of independent chromosome segments between the reference animals and selection candidates.

**Results:**

When the purebred-crossbred correlation was 0.75, the accuracy was highest for a two-way crossbred reference population but similar for purebred and four-way crossbred reference populations, for all reference population sizes. When the purebred-crossbred correlation was 0.5, a purebred reference population always resulted in the lowest accuracy. Among the different crossbred reference populations, the accuracy was slightly lower when more multiplication steps were used to create the crossbreds. In general, the benefit of crossbred reference populations increased when the size of the reference population increased. All predicted accuracies overestimated their corresponding empirical accuracies, but the different scenarios were ranked accurately when the reference population was large.

**Conclusions:**

The benefit of a crossbred reference population becomes larger when the crossbred population is more related to the purebred selection candidates, when the purebred-crossbred correlation is lower, and when the reference population is larger. The purebred-crossbred correlation and reference population size interact with each other with respect to their impact on the accuracy of genomic estimated breeding values.

## Background

In pig and poultry production, the production animals are generally three- or four-way crossbreds. The main reasons for crossbreeding are to benefit from heterosis and breed complementarity, and to be flexible in creating different products for different markets [1–3]. To improve the performance of future production animals, selection takes place in each of the purebred parent lines. Before the genomics era, this selection was often based on purebred performance, because tracing the pedigree of the crossbred production animals back to their purebred ancestors is challenging [4]. However, purebred performance is genetically different from crossbred performance, because purebreds and crossbreds are generally raised in different environments [4–6] and have different genetic backgrounds [7–9]. It has been shown that the genetic correlation between purebred and crossbred performance, known as the purebred-crossbred correlation (${r}_{pc}$), differs substantially from 1 for some traits [10–12]. Using currently available genomic data, linking the crossbred animals and the purebred selection candidates is easier, which facilitates selection of purebred animals for crossbred performance. For example, genotyping can be used to set up a crossbred reference population for genomic prediction, where the genotypes and phenotypes of crossbreds are used to predict breeding values for crossbred performance for a set of genotyped purebred selection candidates [4, 13]. The lower the ${r}_{pc}$ is, the greater is the benefit expected from using crossbred information [10, 14, 15].

A potential limitation of using a crossbred reference population is the relatively weak genetic relatedness between the crossbreds and the purebred selection candidates. The accuracy of genomic prediction is strongly affected by the genetic relatedness between the reference population and the selection candidates [16–18], i.e. the more closely they are related, the higher is the accuracy of genomic prediction. With a purebred reference population, the purebred parents of the selection candidates and their contemporaries can be used as reference animals, resulting in high relatedness between reference and selection animals. In contrast, with a four-way crossbred reference population, the most closely related purebred relatives of the crossbred reference animals are their grandparents, and these purebreds are also the great-grandparents of the selection candidates when the generation interval is the same in purebreds and crossbreds. Hence, in that situation, the reference animals and selection candidates are separated by five generations. Those relationships become even more distant when the production pyramid contains multiplication steps [6, 19]. To date, the effect of the distance in genetic relationship between a crossbred reference population and the purebred selection candidates on the benefit of a crossbred reference population has not been studied.

When designing a breeding program, it is important to be able to rank the expected genetic progress of different scenarios before collecting the data in order to select the most optimal reference population. For this purpose, different deterministic prediction equations for the accuracy of genomic prediction have been derived [20–23]. However, their ability to rank different purebred or crossbred reference populations correctly, in terms of the achieved accuracy of predictions of crossbred performance, has not been investigated.

The aim of this study was to investigate the benefit of using a crossbred reference population for genomic prediction of purebred animals for crossbred performance for: (1) different levels of relatedness between the crossbred reference population and purebred selection candidates, (2) different levels of ${r}_{pc}$, and (3) different reference population sizes. In addition, we investigated the ability to rank the prediction accuracy of different scenarios correctly based on deterministic prediction equations to predict the accuracy of crossbred performance of purebred selection candidates. We simulated a crossbred breeding program with 0, 1 or 2 multiplication steps to generate the crossbreds, and compared the accuracy of genomic prediction for crossbred performance in one generation using either a purebred or a crossbred reference population.

## Methods

**Fig. 1:**
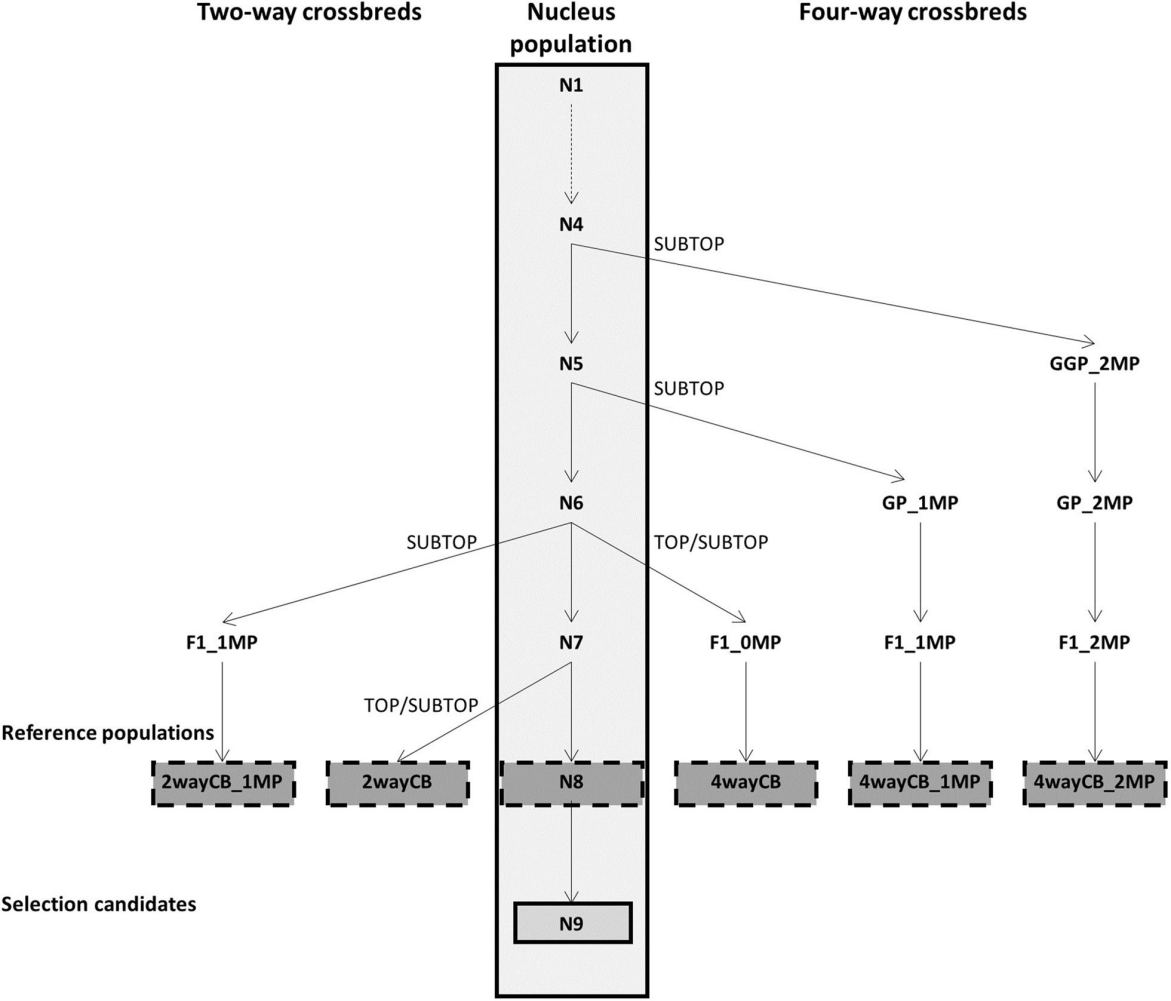
General overview of the crossbred breeding program

We simulated a crossbred breeding program in pigs, where selection in generations 1 through 8 of the nucleus population was based on purebred performance using genomic best linear unbiased prediction (GBLUP) (Fig. 1). From this nucleus population, different types of crossbreds were generated, namely two-way crossbreds with 0 (2wayCB) or 1 (2wayCB_1MP) multiplication step, and four-way crossbreds with 0 (4wayCB), 1 (4wayCB_1MP) or 2 (4wayCB_2MP) multiplication steps. To produce the crossbreds, the second best (SUBTOP) males from the nucleus population were used, i.e. the best purebred males that were not used as parents in the nucleus population. In addition, the scenarios without multiplication steps were also applied using the best (TOP) males to produce crossbreds (2wayCB_TOP and 4wayCB_TOP), i.e., the same males as those that were used to breed the next generation of the nucleus population. Then, each of the resulting crossbred populations was used as reference population to predict genomic estimated breeding values (GEBV) for crossbred performance of purebred nucleus animals in generation 9. The crossbred populations differed in the number of generations that separated them from the purebred selection candidates in generation 9 (Table 1).

**Table 1:** Overview of purebred ancestors of crossbred populations and the number of generations between the crossbred populations and purebred selection candidates

Crossbred population	Purebred ancestors of crossbred animals		Generations difference with selection candidates
	Generation	SUBTOP/TOP	
2wayCB_TOP	7	TOP	3
2wayCB	7	SUBTOP	3
2wayCB_1MP	6	SUBTOP	5
4wayCB_TOP	6	TOP	5
4wayCB	6	SUBTOP	5
4wayCB_1MP	5	SUBTOP	7
4wayCB_2MP	4	SUBTOP	9

Genotypes were simulated for all animals in the nucleus population. For crossbred animals, only the alleles coming from the sire (two-way crossbred) or the paternal grand sire (four-way crossbred) were simulated. This implicitly assumes that the line-origin of the alleles in the crossbreds could be traced back without error and that genomic information from the other lines was not helpful to predict breeding values for the line of interest, as generally observed in practice [24, 25]. The alleles coming from the other lines were set to missing, and their contributions to the phenotypes were simulated as for a polygenic trait.

### Population structure

**Fig. 2:**
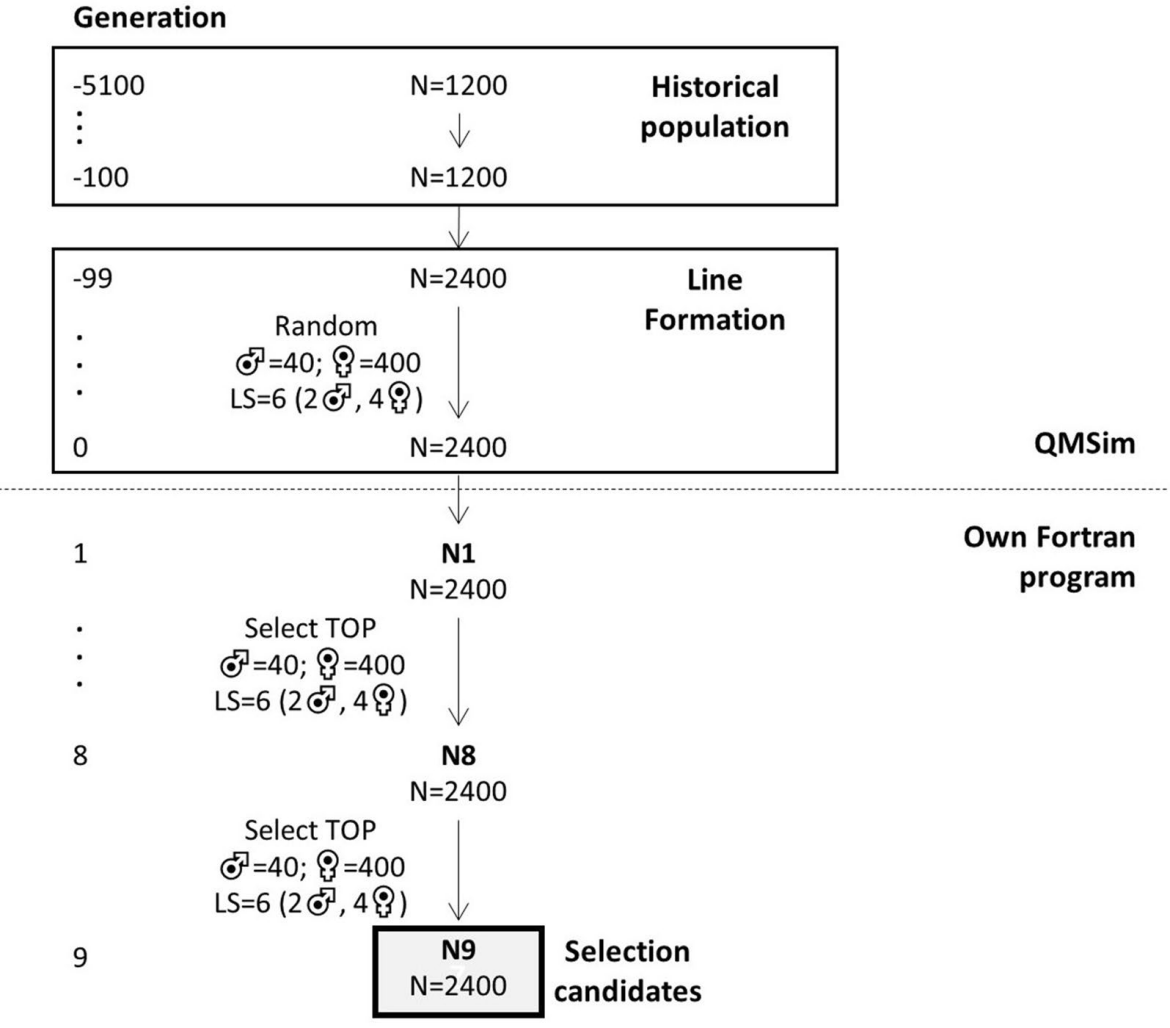
Schematic overview of the historical and nucleus populations

To simulate genotypes of the nucleus population, we started by simulating a historical population (Fig. 2) using the QMSim software [26]. This historical population consisted of 1200 (600 males and 600 females) animals per generation, that were randomly selected and mated for 5000 discrete generations (generation -5100 to -100). From the last historical generation, 40 males and 400 females were randomly selected and mated to create a population that was randomly mated for 100 discrete generations (generations -100 to 0) by randomly selecting 40 males and 400 females to create the next generation, with 6 progeny per female (2 males and 4 females). The formation of this population mimicked the bottleneck that pigs experienced during breed formation [27], and was used to generate linkage disequilibrium on the genome.

From generation 0, 40 males and 400 females were randomly selected and mated to become the parents of the purebred nucleus population, which is referred to as the base generation. In the next nine generations, the best (TOP) 40 males and 400 females in each generation were selected and randomly mated with a mating ratio of 1:10 to generate the next nucleus generation using a litter size of 6 (2 males and 4 females). Selection was based on GEBV for purebred performance using GBLUP [28] implemented with the MiXBLUP software [29]. This GBLUP analysis included all animals from generation 0 onwards. The genomic relationship matrix was calculated using Method 1 of VanRaden [30] and the allele frequencies in the base generation. Variance components were fixed to the simulated variance components in the base generation.

From the nucleus population, 80 SUBTOP or TOP males were selected to produce the different crossbred populations (Table 1 and Fig. 1). In the case of a multiplication step, 800 SUBTOP females were selected from the nucleus population as well. In the other multiplication and crossbreeding steps to generate the crossbred population, 80 males and 800 females were selected randomly. In all steps, mating was at random with a mating ratio of 1:10 and litter size was set to 3 (1 male and 2 females) to keep the same number of animals in the purebred and crossbred reference populations.

### Genome size

The simulated genome consisted of 10 chromosomes of 1 Morgan each. On each chromosome, 20,000 randomly spaced loci were simulated with a recurrent mutation rate of 0.00005 in the historical population. At the end of the historical population, loci with a minor allele frequency (MAF) higher than 0.01 were selected and mutation rate was set to zero.

The parameter settings of the simulated population resulted in a weak U-shaped allele frequency distribution for the loci that segregated (MAF > 0) in the base generation, from which 2000 causal loci and 20,000 markers were selected. The causal loci were selected randomly from the segregating loci. After sampling causal loci, markers were selected by dividing the remaining loci into 100 bins based on allele frequency (i.e., allele frequencies of bin 1 ranged from 0 to 0.01, of bin 2 from 0.01 to 0.02, etc.) and sampling 200 loci as markers from each bin. This resulted in a uniform allele frequency distribution for markers, as is the case generally for commercially available marker chips [31–33].

### Phenotypes

In the base generation, allele substitution effects were assigned to each of the causal loci for both purebred and crossbred performance, assuming that these two traits are correlated. Therefore, allele substitution effects were sampled from a multivariate normal distribution, with a mean of 0 and a standard deviation of 1. The correlation between the allele substitution effects for purebred and crossbred performance was set to 0.75 or 0.5, which represents the ${r}_{pc}$ in this simulation. For purebred animals, allele substitution effects were multiplied by the allele counts of the causal loci (0, 1, or 2) and summed over loci to calculate the true breeding value (TBV) of the animals for both traits. The genetic variance for purebred performance (${{\sigma }_{A}}_{PB}^{2}$) and crossbred performance (${{\sigma }_{A}}_{CB}^{2}$) was computed as the variance of the TBV of the corresponding trait across animals in the base generation.

For purebred animals, only phenotypes for purebred performance were simulated by summing the TBV and an environmental effect that was randomly sampled from a normal distribution with a mean of 0 and a variance equal to ${{\sigma }_{E}}_{PB}^{2}=4*{{\sigma }_{A}}_{PB}^{2}$, such that heritability of the trait was 0.2.

To simulate phenotypes of crossbred animals for crossbred performance, first, haploid genotypes were simulated for the two-way crossbred animals, which consisted of only one allele for each locus coming from their sire by generating gametes produced by the sire. The allele counts of these haploid genotypes were multiplied by the corresponding allele substitution effects for crossbred performance to calculate the true gametic value (TGV) coming from the sire (${\mathrm{TGV}}_{\mathrm{sire}}$). The TGV coming from the dam (${\mathrm{TGV}}_{\mathrm{dam}}$) was simulated as for a polygenic trait. First, a ${\mathrm{TBV}}_{\mathrm{dam}}$ was randomly sampled for all dams from $N(0,{{\sigma }_{A}}_{CB}^{2})$. Second, ${\mathrm{TGV}}_{\mathrm{dam}}$ for each two-way crossbred animal was calculated as ${\mathrm{TGV}}_{\mathrm{dam}}={\frac{1}{2}}{\mathrm{TBV}}_{\mathrm{dam}}+\mathrm{MS}$, where $\mathrm{MS}$ is a Mendelian sampling term sampled from $N(0,\frac{1}{4}{{\sigma }_{A}}_{CB}^{2})$. The TBV of the crossbred animal was then calculated as ${\mathrm{TGV}}_{\mathrm{sire}}+{\mathrm{TGV}}_{\mathrm{dam}}$. Phenotypes for crossbred performance of each crossbred animal were then simulated by summing the TBV and an environmental effect that was randomly sampled from $N(0,{{\sigma }_{E}}_{CB}^{2})$, with ${{\sigma }_{E}}_{CB}^{2}=4*{{\sigma }_{A}}_{CB}^{2}$, resulting in a heritability equal to 0.2. Analyses for two-way crossbred animals without multiplication steps were repeated with a heritability of 0.05 for crossbred performance by setting ${{\sigma }_{E}}_{CB}^{2}={19}^{*}{{\sigma }_{A}}_{CB}^{2}$ because, in practice, crossbred animals are kept in more variable environments, which results in larger environmental variance components.

For four-way crossbred animals, on average 25% of the genome originated from the line of interest via the paternal grand sire ($\mathrm{PGS}$) and only this part was simulated with genotypes. This means that the paternal allele was known for on average 50% of the loci of the four-way crossbred animals. The allele counts of those loci were multiplied with the corresponding allele substitution effects to calculate the TGV coming from the $\mathrm{PGS}$ (${\mathrm{TGV}}_{\mathrm{PGS}}$). The other ~ 50% of the paternal haplotype originated from the $\mathrm{PGD}$, and its effect (${\mathrm{TGV}}_{\mathrm{PGD}}$) was simulated as for a polygenic trait. This step of the simulation considered both the proportion of the genome originating from the $\mathrm{PGD}$ for each simulated crossbred animal and the overlap in that part between sibs (see Additional file 1 for details). The ${\mathrm{TGV}}_{\mathrm{dam}}$ for the four-way crossbreds was sampled in the same way as described before for ${\mathrm{TGV}}_{\mathrm{dam}}$ of the two-way crossbreds. The TBV of four-way crossbred animals was calculated as ${\mathrm{TGV}}_{\mathrm{PGS}}+{\mathrm{TGV}}_{\mathrm{PGD}}+{\mathrm{TGV}}_{\mathrm{dam}}$, and phenotypes for crossbred performance were simulated by summing the TBV and an environmental effect, as described above. Again, for four-way crossbred animals without multiplication steps, the analyses were repeated with a heritability of 0.05.

### Size of the reference population

In the default scenario, each generation contained 2400 animals that could be included in the reference population. To investigate the impact of size of the reference population, analyses were repeated by using 9600, 4800, 1200, 600, and 300 instead of 2400 reference animals. The reference population size was increased by generating more offspring in generation 8 with the same mating design (*i.e.,* by creating more full sibs of animals in the reference population). The reference population size was decreased by randomly sampling a proportion of 0.5, 0.25 or 0.125 of the full-sib and half-sib offspring of each sire. Thus, the number of families was kept the same but family sizes were reduced. For the nucleus population, this means that not all parents of the selection candidates were included in the reference populations of 1200, 600 and 300 animals. We used 50 replicates for all scenarios, except, for computational reasons, only 10 replicates were used for the scenarios with 9600 and 4800 crossbred animals in the reference population. Scripts and seeds to simulate the data are in Additional file 2.

### Empirical accuracy

We were interested in the accuracy of GEBV for crossbred performance of purebred selection candidates in generation 9 based on different reference populations. The reference population consisted of all (i.e., 300, 600, 1200, 2400, 4800, or 9600) animals from generation 8 (Fig. 1). GEBV were estimated using GBLUP [28]. Because the reference populations with 300 or 600 animals were too small to accurately estimate the genetic variance, we fixed the genetic variance of the traits (either purebred or crossbred) that originated from the line of interest to its value in the base generation (${{\sigma }_{A}}_{PB}^{2}$ and ${{\sigma }_{A}}_{CB}^{2}$). All other variance components were estimated using ASReml 4.1 [34]. To check the impact of fixing the genetic variance, we also repeated all analyses by estimating all variance components.

For the purebred reference population, we used the following model:

$${\mathbf{y}}_{{\varvec{P}}{\varvec{B}}}=\mu +\mathbf{Z}\mathbf{u}+\mathbf{e},$$

where $\mu$ is a general mean, ${\mathbf{y}}_{{\varvec{P}}{\varvec{B}}}$ is a vector of phenotypes of purebred reference animals, $\mathbf{u}$ is a vector of breeding values for purebred performance of reference animals and selection candidates (${\mathbf{u}\sim N({\mathbf{0}},\mathbf{G}\sigma }_{{A}_{PB}}^{2}$)), $\mathbf{Z}$ is an incidence matrix linking phenotypes to breeding values, and $\mathbf{e}$ is a vector of residuals ($\mathbf{e}\sim N({\mathbf{0}}, \mathbf{I}{\sigma }_{e}^{2}$)). The genomic relationship matrix, $\mathbf{G}$, was calculated following method 1 of VanRaden [30] as: $\mathbf{G}=\frac{\mathbf{M}{\mathbf{M}}^{\mathbf{^{\prime}}}}{\sum 2{p}_{j}(1-{p}_{j})}$, where $\mathbf{M}$ is a centered marker genotype matrix of purebred animals in which, for each animal, allele counts (i.e., 0, 1 or 2) of each locus $j$ were centered by subtracting $2{p}_{j}$, where ${p}_{j}$ is the observed allele frequency of locus $j$ in the base generation.

For the two-way crossbred reference population, the model was:

$${\mathbf{y}}_{{\varvec{2w}}{\varvec{a}}{\varvec{y}}{\varvec{C}}{\varvec{B}}}={\mu +\mathbf{Z}}_{\mathrm{Pat}}{\mathbf{u}}_{\mathrm{Pat}}+\mathbf{L}\mathbf{m}+\mathbf{e},$$

where ${\mathbf{y}}_{{\varvec{2w}}{\varvec{a}}{\varvec{y}}{\varvec{C}}{\varvec{B}}}$ is a vector of phenotypes of two-way crossbred reference animals, ${\mathbf{u}}_{\mathrm{Pat}}$ is a vector of breeding values of crossbred reference animals based on their paternal alleles and breeding values for crossbred performance of purebred selection candidates with incidence matrix ${\mathbf{Z}}_{\mathrm{Pat}}$ and ${{\mathbf{u}}_{\mathrm{Pat}}\sim N({\mathbf{0}},{\mathbf{G}}^{(\mathrm{NP})}\sigma }_{{A}_{CB}}^{2}$), $\mathbf{m}$ is a vector of maternal effects ($\mathbf{m}\sim N({\mathbf{0}},{{\mathbf{I}}_{m}\sigma }_{m}^{2}$) for all dams $m$ with identity matrix ${\mathbf{I}}_{m}$) accounting for the resemblance between full sibs due to the dam, and $\mathbf{L}$ is the corresponding incidence matrix. Note that the maternal effect was fitted with an identity matrix, because no relationship structure was simulated in the dams. The relationship matrix ${\mathbf{G}}^{(\mathrm{NP})}$ for ${\mathbf{u}}_{\mathrm{Pat}}$ was a partial relationship matrix based only on the paternal alleles originating from the nucleus population ($\mathrm{NP}$), which represented 50% of the genome in the two-way crossbreds and was calculated following Sevillano et al*.* [24] as $${\mathbf{G}}^{(\mathrm{NP})}=\left[\begin{array}{cc}\frac{\mathbf{M}{\mathbf{M}}^{\mathbf{^{\prime}}}}{\sum 2{p}_{j}(1-{p}_{j})}& \frac{\mathbf{M}{\mathbf{T}}^{\mathbf{^{\prime}}}}{\sum 2{p}_{j}(1-{p}_{j})}\\ \frac{\mathbf{T}{\mathbf{M}}^{\mathbf{^{\prime}}}}{\sum 2{p}_{j}(1-{p}_{j})}& \frac{\mathbf{T}{\mathbf{T}}^{\mathbf{^{\prime}}}}{\sum 2{p}_{j}(1-{p}_{j})}\end{array}\right]$$, where $\mathbf{T}$ is a centered marker genotype matrix of crossbred animals, with allele counts coming from the nucleus population (i.e., 0 or 1) of each animal centered by subtracting ${p}_{j}$ for each locus. Since the full genome of the purebreds and half of the genome of two-way crossbreds were used to estimate the relationship matrix, average diagonal elements were 1 for purebreds and 0.5 for crossbreds (see Additional file 3: Figure S3.1). The genetic variance due to the dam consisted of between-family variance captured by $\mathbf{m}$, and within-family segregation variance, which was included in $\mathbf{e}$. For this reason, ${\sigma }_{m}^{2}$ and ${\sigma }_{e}^{2}$ were estimated together with the breeding values.

For the four-way crossbred reference population, the model was:

$${\mathbf{y}}_{{\varvec{4w}}{\varvec{a}}{\varvec{y}}{\varvec{C}}{\varvec{B}}}=\mu +{\mathbf{Z}}_{\mathrm{PGS}}{\mathbf{u}}_{\mathrm{PGS}}+{\mathbf{Z}}_{\mathrm{PGD}}{\mathbf{a}}_{\mathrm{PGD}}+\mathbf{L}\mathbf{m}+\mathbf{e},$$

where ${\mathbf{y}}_{{\varvec{4w}}{\varvec{a}}{\varvec{y}}{\varvec{C}}{\varvec{B}}}$ is a vector of phenotypes of four-way crossbred reference animals, ${\mathbf{u}}_{\mathrm{PGS}}$ is a vector of breeding values of crossbred reference animals accounting for the contribution of the $\mathrm{PGS}$ and breeding values for crossbred performance of purebred selection candidates, with incidence matrix ${\mathbf{Z}}_{\mathrm{PGS}}$ and ${{\mathbf{u}}_{\mathrm{PGS}}\sim N({\mathbf{0}},{\mathbf{G}}^{\left(\mathrm{NP}\right)}\sigma }_{{A}_{CB}}^{2})$, ${\mathbf{a}}_{\mathrm{PGD}}$ is a vector of breeding values for crossbred performance of crossbred reference animals accounting for the contribution of the $\mathrm{PGD},$ with incidence matrix ${\mathbf{Z}}_{\mathrm{PGD}}$ and ${\mathbf{a}}_{\mathrm{PGD}}\sim N({\mathbf{0}},{\mathbf{G}}^{(\mathrm{PGD})}{\sigma }_{{A}_{CB,PGD}}^{2}$). Note that the notation for the breeding value coming from the $\mathrm{PGD}$ ($\mathbf{a}$) is different than from the PGS ($\mathbf{u}$), since the breeding value from the PGD was simulated as for a polygenic trait and was not based on genotypes. The relationship matrix ${\mathbf{G}}^{\left(\mathrm{NP}\right)}$ for ${\mathbf{u}}_{\mathrm{PGS}}$ was a partial relationship matrix based only on alleles that originated from the $\mathrm{PGS}$ in the nucleus population, which represented approximately 25% of the genome in the four-way crossbreds, and also included the purebred selection candidates. This matrix was again calculated following Sevillano et al*.* [24] as $${\mathbf{G}}^{(\mathrm{NP})}=\left[\begin{array}{cc}\frac{\mathbf{M}{\mathbf{M}}^{\mathbf{^{\prime}}}}{\sum 2{p}_{j}(1-{p}_{j})}& \frac{\mathbf{M}{\mathbf{T}}^{\mathbf{^{\prime}}}}{\sum 2{p}_{j}(1-{p}_{j})}\\ \frac{\mathbf{T}{\mathbf{M}}^{\mathbf{^{\prime}}}}{\sum 2{p}_{j}(1-{p}_{j})}& \frac{\mathbf{T}{\mathbf{T}}^{\mathbf{^{\prime}}}}{\sum 2{p}_{j}(1-{p}_{j})}\end{array}\right]$$, where allele counts in $\mathbf{T}$ were set to missing for loci for which a crossbred animal did not carry an allele that originated from the nucleus population, which resulted in that locus effectively not contributing to the estimated relationships. Since the full genome of the purebreds and only a quarter of the genome of the four-way crossbreds were used to estimate the relationship matrix, average diagonal elements were 1 for purebreds and 0.25 for crossbreds (see Additional file 3 Figure S3.2). The relationships matrix ${\mathbf{G}}^{(\mathrm{PGD})}$ related to ${\mathbf{a}}_{\mathrm{PGD}}$ was also a partial relationship matrix and was estimated as the proportion of the genome that originated from the $\mathrm{PGD}$ that overlapped between sibs. It included only crossbred animals. The four-way crossbreds differed in the proportion of their genome that originated from the line of interest (*i.e.*, from the $\mathrm{PGS}$), because of segregation in the F1 sires. For this reason, part of the genetic variation in ${\mathbf{u}}_{\mathrm{PGS}}$ among the four-way crossbreds resulted from the variation in the number of genes that originated from the $\mathrm{PGS}$ *vs.* $\mathrm{PGD}$ line (see Additional file 1 for details). Including the ${\mathbf{a}}_{\mathrm{PGD}}$ term enabled the model to capture this variation, since 50% of the genetic variance in the crossbreds was always explained by ${\mathbf{u}}_{\mathrm{PGS}}+{\mathbf{a}}_{\mathrm{PGD}}$. Again,${\sigma }_{m}^{2}$ and ${\sigma }_{e}^{2}$ were estimated together with the breeding values.

For the scenarios with only 300 or 600 crossbred reference animals, the analyses did not include a maternal effect because full sibs were only present for part of the crossbred animals with 600 reference animals, or for none of the crossbred animals with 300 reference animals.

For all scenarios, empirical accuracy of GEBV was estimated as the correlation between the GEBV and the TBV for crossbred performance of the purebred selection candidates in generation 9. Moreover, we investigated the bias of the scale of the GEBV by estimating the regression coefficient of the TBV on the GEBV. For the crossbred reference populations, GEBV were unbiased when this regression coefficient was 1. For the purebred reference populations, this regression coefficient was equal to $\frac{Cov({\mathbf{T}\mathbf{B}\mathbf{V}}_{\mathrm{CB}},{\mathbf{G}\mathbf{E}\mathbf{B}\mathbf{V}}_{\mathrm{PB}})}{Var({\mathbf{G}\mathbf{E}\mathbf{B}\mathbf{V}}_{\mathrm{PB}})}$, where ${\mathbf{T}\mathbf{B}\mathbf{V}}_{\mathrm{CB}}$ is a vector of TBV for crossbred (CB) performance and ${\mathbf{G}\mathbf{E}\mathbf{B}\mathbf{V}}_{\mathrm{PB}}$ is a vector of GEBV for purebred (PB) performance of purebred selection candidates. We can write ${\mathbf{T}\mathbf{B}\mathbf{V}}_{\mathrm{CB}}$ as ${r}_{pc}*{\mathbf{T}\mathbf{B}\mathbf{V}}_{\mathrm{PB}}+\mathbf{e}$, where $\mathbf{e}$ is a vector of residual terms that are independent of the ${\mathbf{G}\mathbf{E}\mathbf{B}\mathbf{V}}_{\mathrm{PB}}$, because variance components were fixed to variance components in the base generation, for which ${{\sigma }_{A}}_{PB}^{2}$ and ${{\sigma }_{A}}_{CB}^{2}$ were the same. Hence, $\frac{Cov({\mathbf{T}\mathbf{B}\mathbf{V}}_{\mathrm{CB}},{\mathbf{G}\mathbf{E}\mathbf{B}\mathbf{V}}_{\mathrm{PB}})}{Var({\mathbf{G}\mathbf{E}\mathbf{B}\mathbf{V}}_{\mathrm{PB}})}={r}_{pc}\frac{Cov({\mathbf{T}\mathbf{B}\mathbf{V}}_{\mathrm{PB}},{\mathbf{G}\mathbf{E}\mathbf{B}\mathbf{V}}_{\mathrm{PB}})}{Var({\mathbf{G}\mathbf{E}\mathbf{B}\mathbf{V}}_{\mathrm{PB}})}$. Given that the expectation for $\frac{Cov({\mathbf{T}\mathbf{B}\mathbf{V}}_{\mathrm{PB}},{\mathbf{G}\mathbf{E}\mathbf{B}\mathbf{V}}_{\mathrm{PB}})}{Var({\mathbf{G}\mathbf{E}\mathbf{B}\mathbf{V}}_{\mathrm{PB}})}$ is 1, the expectation for $\frac{Cov({\mathbf{T}\mathbf{B}\mathbf{V}}_{\mathrm{CB}},{\mathbf{G}\mathbf{E}\mathbf{B}\mathbf{V}}_{\mathrm{PB}})}{Var({\mathbf{G}\mathbf{E}\mathbf{B}\mathbf{V}}_{\mathrm{PB}})}$ is ${r}_{pc}$ when GEBV are unbiased.

### Predicted accuracy

We also investigated the potential to predict the accuracy of the GEBV for crossbred performance of purebred selection candidates. For the crossbred reference populations, this accuracy was predicted as [20, 35]:

$${r}_{IH}=\sqrt{\frac{N{h}^{2}}{N{h}^{2}+{M}_{e}}},$$

where $N$ is the number of animals in the reference population, ${h}^{2}$ is the heritability of the trait in the reference population (*i.e.,* Bulmer-equilibrium ${h}^{2}$), and ${M}_{e}$ is the effective number of independent chromosome segments between the reference population and selection candidates. Parameter ${M}_{e}$ was estimated from the data as the reciprocal of the variance in genomic relationships between the reference population and selection candidates [22, 36, 37]. Similarly, we used the reciprocal of the variance of partial relationships between the reference population and selection candidates to compute the ${M}_{e}$ between a crossbred reference population and purebred selection candidates, thereby considering only alleles that originated from the nucleus population.

For the purebred reference population, accuracy was predicted as [22, 23]:

$${r}_{IH}={r}_{pc}\sqrt{\frac{N{h}^{2}}{N{h}^{2}+{M}_{e}}},$$

where ${r}_{pc}$ is included because the reference population is measured for purebred performance whereas the prediction refers to crossbred performance. A single value of ${M}_{e}$ was used for all reference population sizes, and this value was estimated using a reference population size of 2400 animals.

Based on these prediction equations, we created an R script (available in Additional file 4) that visualized the difference in predicted accuracy of a purebred versus a crossbred reference population for a range of ${r}_{pc}$ values and reference population sizes by means of a contour plot. The input parameters for this script are the ${M}_{e}$ between the purebred or crossbred reference populations and purebred selection candidates, and the heritabilities for purebred and crossbred performance.

## Results

### Population properties

**Fig. 3:**
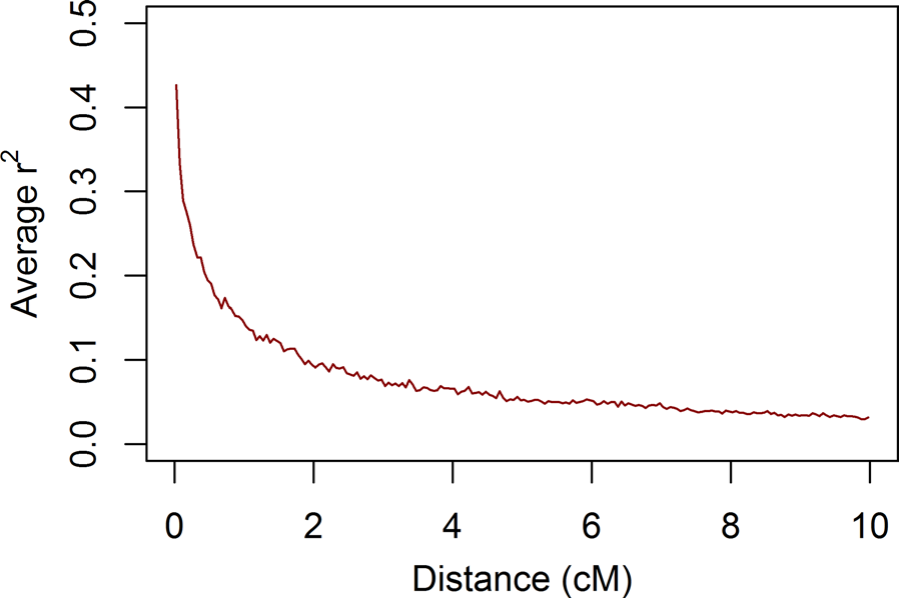
Average strength of linkage disequilibrium (${r}^{2}$) between loci as a function of distance for one replicate

**Fig. 4:**
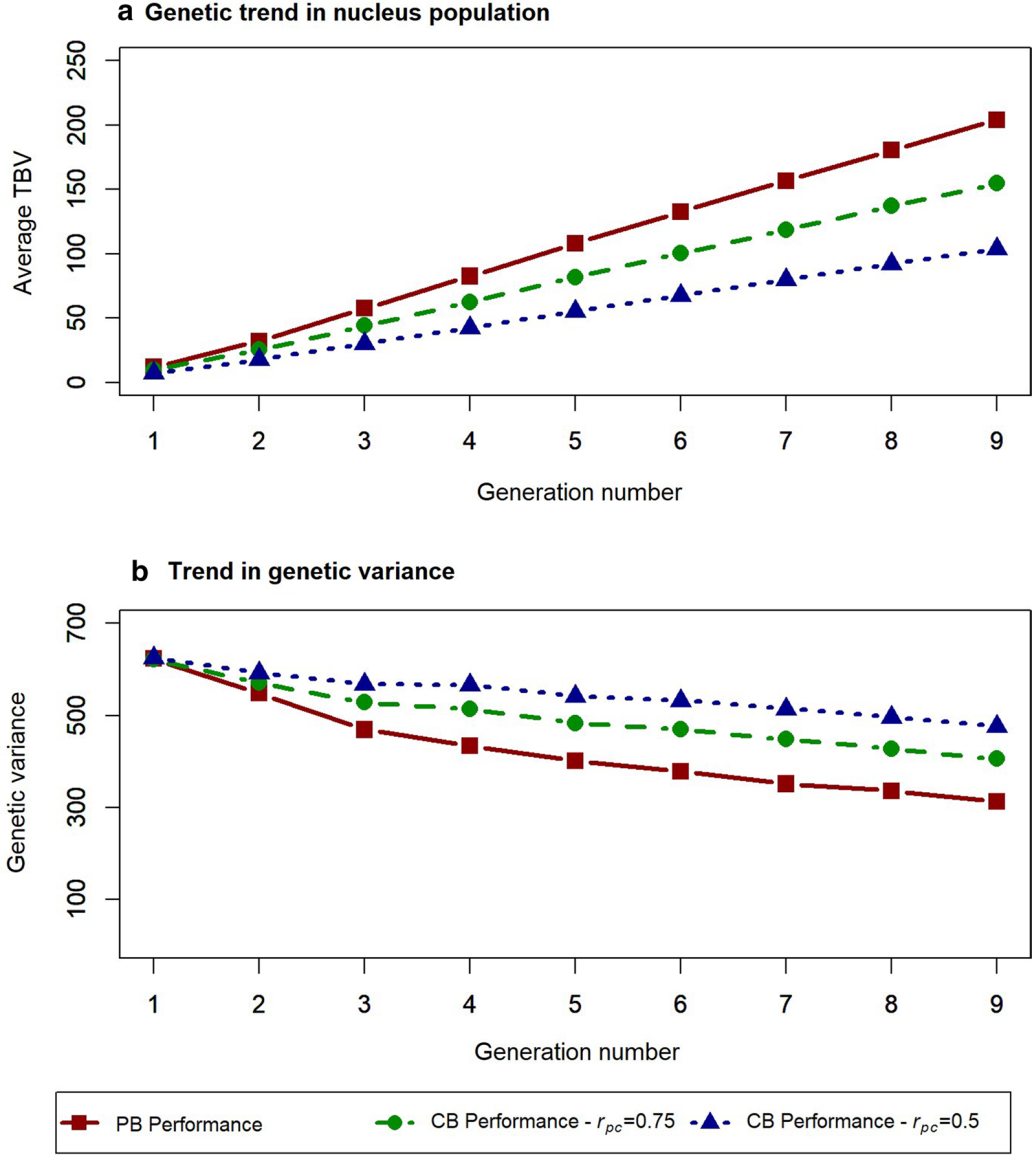
Genetic trend (**a**) and trend in genetic variance (**b**) in the nucleus population with direct selection for purebred (PB) performance. Genetic trend is based on average true breeding value (TBV) across generations for purebred and crossbred (CB) performance, for two levels of the correlation between purebred and crossbred performance (${r}_{pc}$). Direct selection is on purebred performance, with parameters for crossbred performance changing indirectly as a result of ${r}_{pc}$

The linkage disequilibrium (LD) pattern in the simulated nucleus population (Fig. 3) was comparable to that reported for pig populations [38, 39]. In the nucleus population, selection was on purebred performance and, thus, the increase in TBV and the reduction in genetic variance were larger for purebred performance than for crossbred performance (Fig. 4). As expected, the change in average TBV and the reduction in genetic variance for the crossbred traits were ~ 75 and 50% of those of the purebred trait with ${r}_{pc}$ equal to 0.75 and 0.5, respectively. The largest reductions in genetic variance (~ 30%) occurred in the first three generations as a result of the Bulmer effect [40]. Thereafter, an additional 20% of the genetic variance for purebred performance was lost because some genetic variants were driven to fixation, while no new mutations were simulated. Altogether, these results indicate that the simulated population is a realistic representation of a pig population with a history of selection for purebred performance.

### Empirical accuracies for different reference populations

**Fig. 5:**
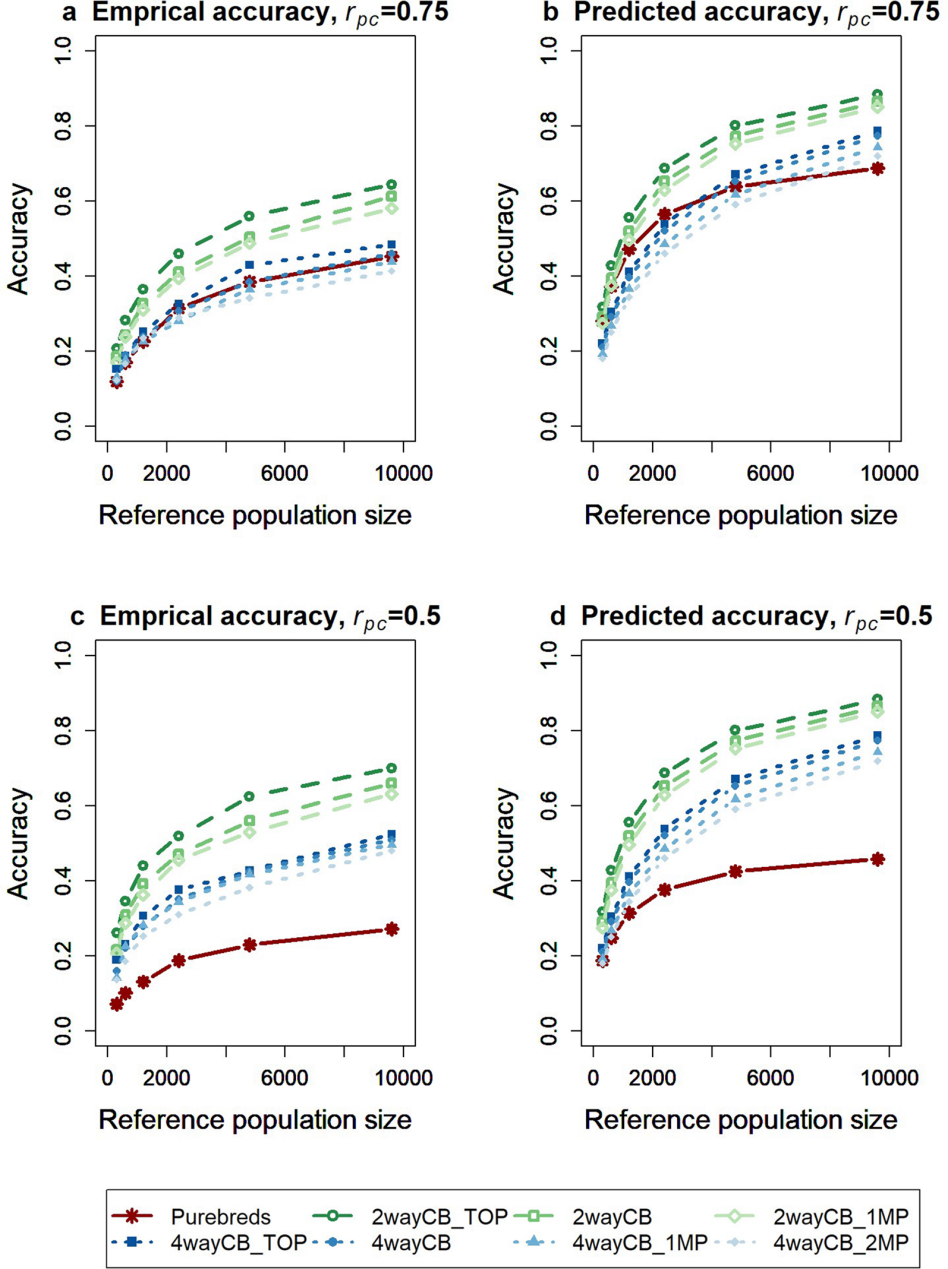
Average empirical (**a** and **c**) and predicted accuracy (**b** and **d**) of estimated breeding values of purebred selection candidates for crossbred performance. The reference population consisted of purebreds (PB), two-way crossbreds (CB) derived from the same sires as used in the nucleus (2wayCB_TOP), different sires with zero (2wayCB) or one multiplication step (2wayCB_1MP), four-way crossbreds derived from the same sires as used in the nucleus population (4wayCB_TOP), different sires with zero (4wayCB), one (4wayCB_1MP), or two multiplication steps (4wayCB_2MP). The purebred-crossbred correlation was equal to 0.75 (**a** and **b**) or 0.5 (**c** and **d**). Heritability was 0.2 in the purebred and crossbred populations. Averages were calculated across 50 replicates

The average empirical accuracies of GEBV for crossbred performance of purebred selection candidates are in Fig. 5a and c, with genetic variance components fixed to the simulated value. As expected, all accuracies increased as the size of the reference population increased. Average empirical accuracies did not change when all genetic variance components were estimated [see Additional file 5 Figure S5.1].

When ${r}_{pc}$ was set to 0.75 (Fig. 5a), accuracy was highest for the two-way crossbred reference populations for all reference population sizes. Among the different two-way crossbred populations, accuracy was highest for the population that was most closely related to the purebred selection candidates (2wayCB_TOP) and lowest for the population that was least closely related to the purebred selection candidates (2wayCB_1MP). The difference in accuracy between the three two-way crossbred reference populations was ~ 0.07 with 2400 animals in the reference population (2wayCB_TOP: accuracy = 0.46; 2wayCB_1MP: accuracy = 0.39). Accuracies were roughly similar for the purebred and four-way crossbred reference populations, with higher accuracies for four-way crossbred reference populations that were more closely related to the selection candidates. Although the difference in relatedness with the selection candidates was larger among the four-way crossbred populations than among two-way crossbred reference populations, the difference in accuracy was smaller among the four-way crossbred populations (only ~ 0.04 with 2400 animals in the reference population; 4wayCB_TOP: accuracy = 0.33; 4wayCB_2MP: accuracy = 0.29).

As expected, the accuracies of GEBV of purebred selection candidates for crossbred performance that was obtained with the crossbred reference populations were not affected by ${r}_{pc}$, but the accuracy obtained with a purebred reference population was about one third lower with ${r}_{pc}$ equal to 0.5 than with ${r}_{pc}$ equal to 0.75. Therefore, when the ${r}_{pc}$ was set to 0.5 (Fig. 5c), the purebred reference populations always resulted in the lowest accuracy. Remarkably, the accuracy obtained with a two-way crossbred reference population was always slightly more than double the accuracy obtained with a purebred reference population. This is probably related to the Bulmer effect due to selection for purebred performance in generations 1 through 8, which resulted in a larger reduction in the genetic variance for the purebred trait than for the crossbred trait.

### Predicted accuracies for different reference populations

**Fig. 6:**
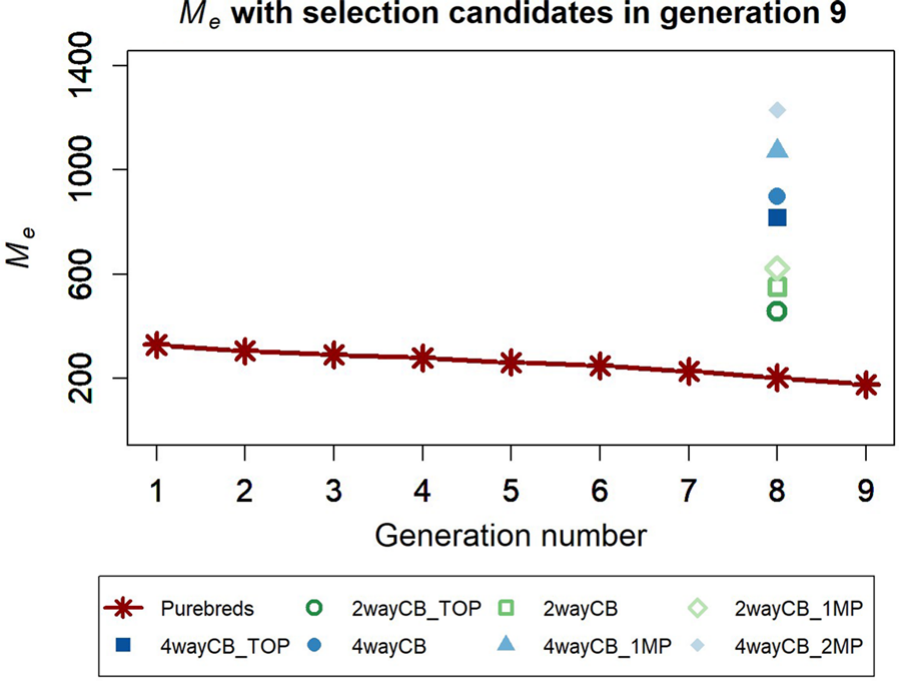
Estimate of the effective number of independent chromosome segments (${M}_{e}$) between the purebred selection candidates and different reference populations. The reference population consisted of purebreds (PB), two-way crossbreds (CB) derived from the same sires as used in the nucleus (2wayCB_TOP), different sires with zero (2wayCB) or one multiplication step (2wayCB_1MP), four-way crossbreds derived from the same sires as used in the nucleus population (4wayCB_TOP), different sires with zero (4wayCB), one (4wayCB_1MP), or two multiplication steps (4wayCB_2MP). The purebred-crossbred correlation was equal to 0.75 (**a** and **b**) or 0.5 (**c** and **d**). Averages were calculated across 50 replicates

In order to predict the accuracies of GEBV of purebred selection candidates for crossbred performance, the ${M}_{e}$ between the reference population and selection candidates had to be estimated (Fig. 6). For illustration, ${M}_{e}$ was also calculated for each generation of the nucleus population with the selection candidates in generation 9, which showed that ${M}_{e}$ increased when the reference animals were less related to the selection candidates. For example, the ${M}_{e}$ between animals in generations 1 and 9 was larger (328) than between animals in generation 8 and 9 (202). This indicates that animals from generation 8 are more useful to predict breeding values for animals from generation 9 than animals from generation 1.

For the crossbred reference populations, ${M}_{e}$ was estimated only between crossbred animals in generation 8 and purebred selection candidates in generation 9. The results show a smaller ${M}_{e}$ between the two-way crossbreds and the selection candidates than between the four-way crossbreds and the selection candidates. Moreover, within the two-way and four-way crossbred reference populations, ${M}_{e}$ was smaller for populations that were more closely related to the purebred selection candidates, which matches with the differences in empirical accuracies. The ${M}_{e}$ between the two-way crossbreds and purebred selection candidates (551) was approximately twice that for the purebred population, which was the same number of generations removed from the selection candidates (generation 6, ${M}_{e}$ =248). The ${M}_{e}$ between the four-way crossbred reference population and purebred selection candidates (898) was approximately four times larger than the ${M}_{e}$ for the purebred population, which was the same number of generations removed from the selection candidates (generation 4, ${M}_{e}$ = 278).

The estimated ${M}_{e}$ values were used to predict the accuracy of GEBV of purebred selection candidates for crossbred performance for the different scenarios (Fig. 5b and d). The predicted accuracies all exceeded the empirical accuracies. This is probably because the predictions assume that all the genetic variance in the selection candidates was explained by the markers, which is too optimistic. The overall trend in predicted accuracies was, however, very similar to the trend in empirical accuracies, which resulted in a similar ranking of the scenarios at each size of the reference population. The only exception was for the purebred reference population, for which the empirical accuracy decreased faster than the predicted accuracy for reference population sizes smaller than 2400 animals. This is probably because not all parents of the selection candidates were included in the smaller purebred reference populations, which reduces the genetic relationships between the reference and selection animals, thereby negatively affecting the accuracy. This is in contrast to the predicted accuracy, for which the same ${M}_{e}$ value was used for all reference population sizes, assuming that the genetic link was constant for decreasing reference population sizes.

**Fig. 7:**
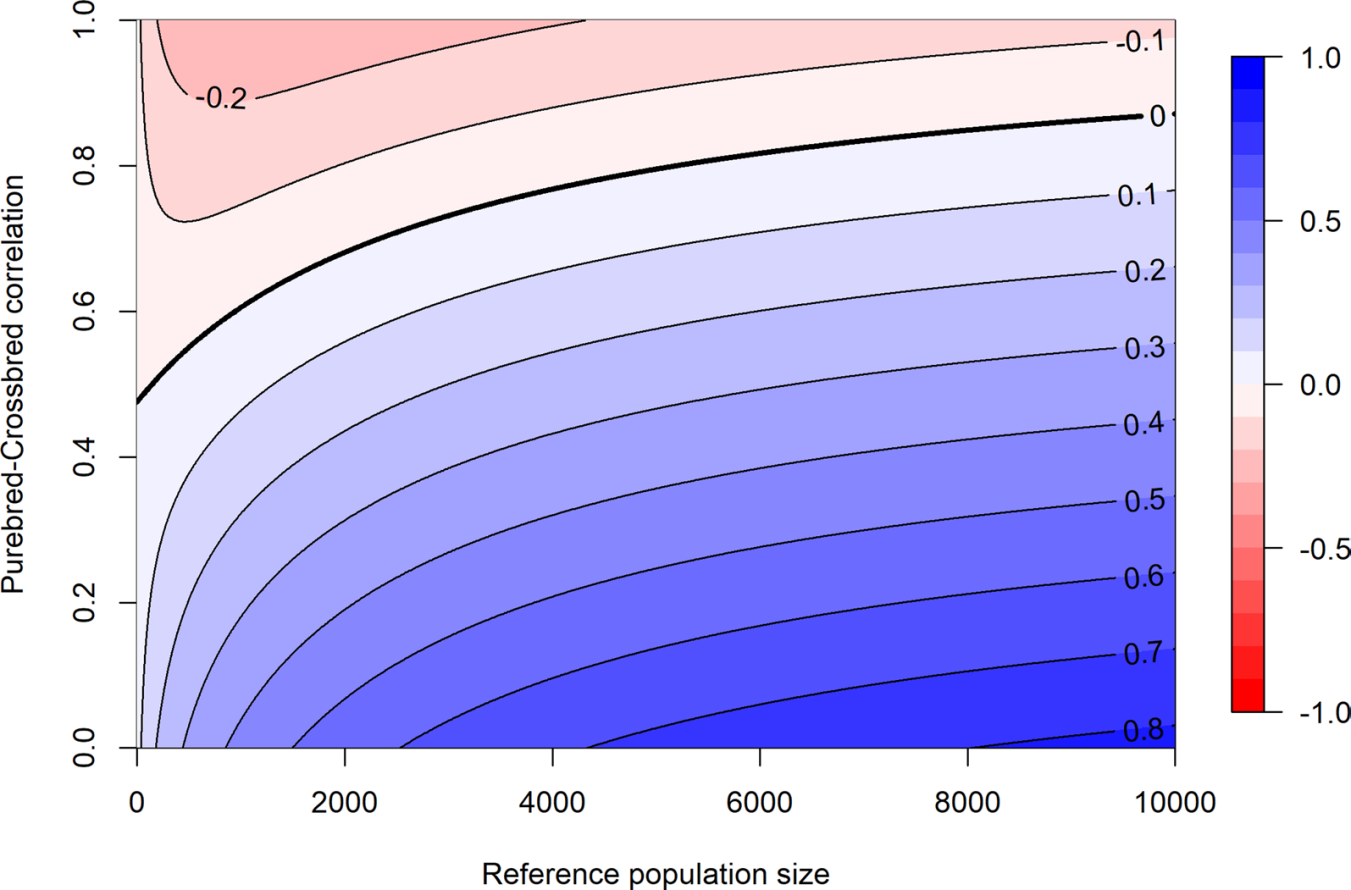
Expected benefit in accuracy of a four-way crossbred over a purebred reference population. The accuracy of a four-way crossbred reference population minus the accuracy obtained with an equally sized purebred reference population across different reference population sizes and purebred-crossbred correlations. Blue color indicates a benefit for the crossbred reference population, and red color indicates a benefit for the purebred reference population. Values for ${M}_{e}$ were set equal to the estimated values within this study (202 for purebreds and 898 for crossbreds), and the heritability was set to 0.2

Figure 7 shows the predicted difference in accuracy when using a four-way crossbred reference population versus a purebred reference population, using the estimated ${M}_{e}$ values and a heritability of 0.2. This figure clearly shows an interaction between the size of the reference population and ${r}_{pc}$ with respect to their impact on the accuracy of GEBV. When ${r}_{pc}$ is 0.6, for example, the purebred and crossbred reference populations are expected to give similar accuracies for a reference population size of 1000, whereas the crossbred reference population is expected to result in an accuracy that is 0.2 higher for a reference population size of 10,000.

### Lower heritability for crossbred animals

**Fig. 8:**
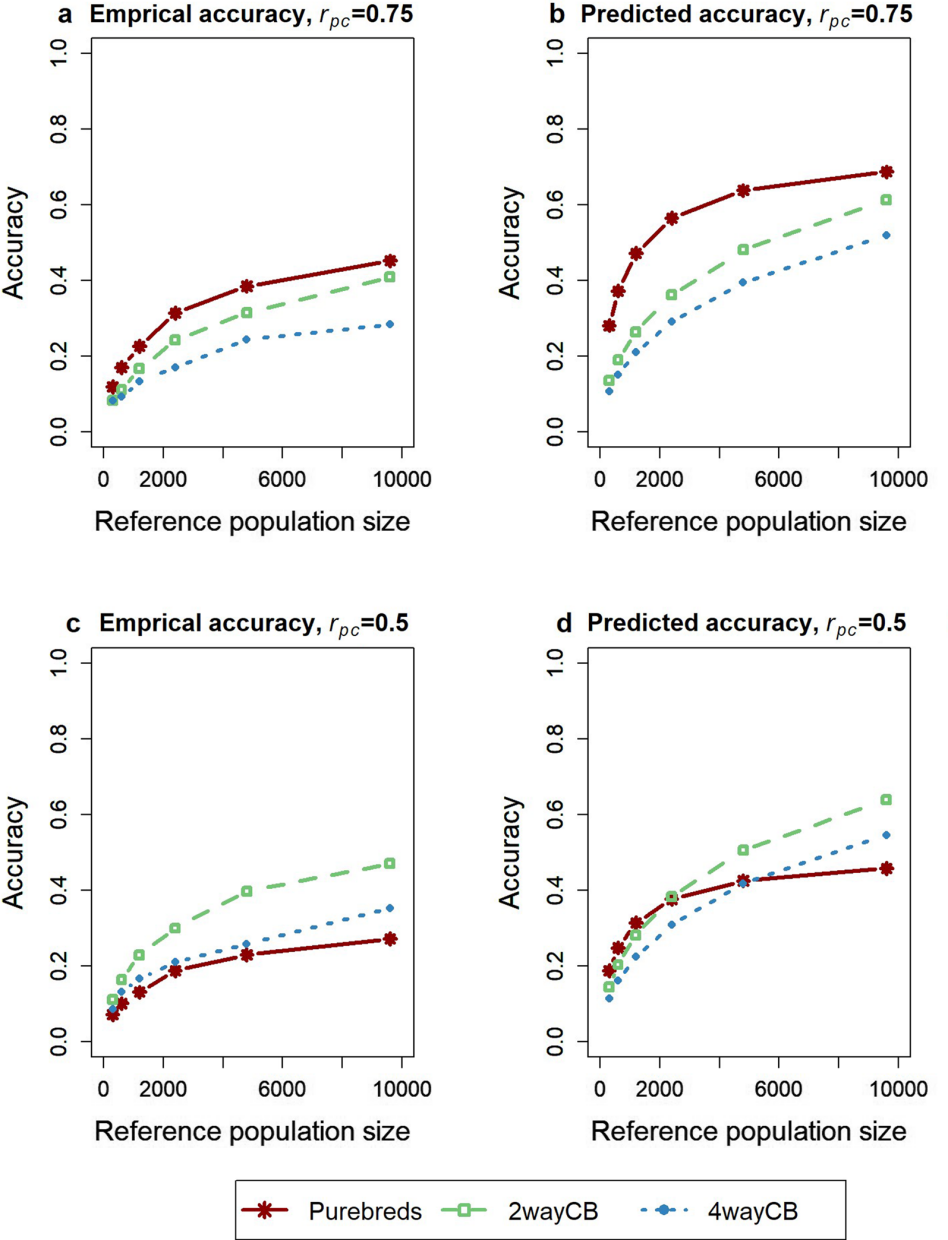
Average empirical (**a** and **c**) and predicted accuracy (**b** and **d**) of estimated breeding values of purebred selection candidates for crossbred performance with a lower heritability in the crossbred populations. The reference population consisted of purebreds (PB), two-way crossbreds (CB) derived from different sires as used in the nucleus with zero multiplication steps (2wayCB), four-way crossbreds derived from different sires as used in the nucleus with zero multiplication steps (4wayCB). The purebred-crossbred correlation was equal to 0.75 (**a** and **b**) or 0.5 (**c** and **d**). Heritability was 0.2 in the purebred population, and 0.05 in the crossbred populations. Averages were calculated across 50 replicates

Results with a lower heritability of 0.05 in the crossbred population compared to a heritability of 0.20 in the purebred population are in Fig. 8. When ${r}_{pc}$ was 0.75, the purebred reference population outperformed the crossbred reference population for all reference population sizes. This was different when the ${r}_{pc}$ was 0.5, for which both crossbred reference populations outperformed the purebred reference population for all reference population sizes. The ranking of the scenarios was accurately predicted when ${r}_{pc}$ was 0.75. However, when ${r}_{pc}$ was 0.5, the ranking differed for small sizes of the reference population, which, to a lesser extent, was also the case when the heritability was the same in the purebred and crossbred populations.

### Bias of estimated breeding values

Table 2 shows estimates of the regression coefficients of the TBV on the GEBV, which is a measure of bias of the scale of the GEBV. Note that, for a purebred reference population, the expected value of the regression coefficient was equal to ${r}_{pc}$ because a purebred reference population resulted in GEBV for purebred performance. For a crossbred reference population, the expected value of the regression coefficient equals 1. All regression coefficients were smaller than their expected value, indicating that the variance in GEBV was higher than expected. For all scenarios, the regression coefficients decreased when the size of the reference population decreased, indicating greater bias for smaller reference populations. Moreover, with a crossbred reference population, the regression coefficients were lower when ${r}_{pc}$ was higher.

**Table 2:** Regression coefficients of true breeding values on genomic estimated breeding values of purebred selection candidates for crossbred performance for different reference populations

Scenario	Reference population size					
	9600	4800	2400	1200	600	300
Purebred-crossbred correlation is 0.75						
Purebred	0.65	0.59	0.53	0.42	0.36	0.29
2wayCB_TOP	0.90	0.86	0.81	0.75	0.71	0.65
2wayCB	0.89	0.84	0.79	0.75	0.69	0.67
2wayCB_1MP	0.87	0.84	0.76	0.72	0.69	0.65
4wayCB_TOP	0.79	0.82	0.75	0.71	0.68	0.75
4wayCB	0.78	0.79	0.73	0.70	0.73	0.59
4wayCB_1MP	0.76	0.74	0.70	0.70	0.71	0.70
4wayCB_2MP	0.74	0.73	0.75	0.77	0.70	0.69
Purebred-crossbred correlation is 0.5						
Purebred	0.42	0.38	0.34	0.26	0.23	0.19
2wayCB_TOP	0.94	0.94	0.92	0.94	0.90	0.87
2wayCB	0.94	0.94	0.91	0.90	0.90	0.81
2wayCB_1MP	0.96	0.91	0.90	0.87	0.87	0.83
4wayCB_TOP	0.89	0.85	0.89	0.90	0.87	0.98
4wayCB	0.88	0.86	0.86	0.86	0.87	0.85
4wayCB_1MP	0.89	0.91	0.87	0.91	0.96	0.80
4wayCB_2MP	0.89	0.83	0.86	0.87	0.84	0.83

Standard errors of the regression coefficient were ranging between 0.022 and 0.080, with an average standard error of 0.036

Biases were smaller when the genetic variance was estimated and the reference population was large (2400 or more animals; see Additional file 5 Table S5.1). However, when the reference population was smaller, the number of animals was too small to accurately estimate the genetic variance, resulting in estimated genetic variance components converging to zero, which caused the estimated regression coefficients to become extremely large.

## Discussion

The first aim of this study was to investigate the benefit of using a crossbred reference population for genomic prediction of purebred selection candidates for crossbred performance, for (1) different levels of relatedness between the crossbred reference population and purebred selection candidates, (2) different levels of ${r}_{pc}$, and (3) different reference population sizes. Results showed that the benefit of a crossbred reference population is larger when the crossbred population is more related to the purebred selection candidates, ${r}_{pc}$ is lower, and the reference population is larger. Moreover, our results showed that the accuracy based on a crossbred reference population is higher when the same (TOP) sires are used to generate purebred and crossbred offspring compared to using the SUBTOP sires to generate crossbred offspring. This gain was larger for two-way than four-way crossbreds, because of the relative larger increase in relatedness for the two-way crossbreds.

A crossbred reference population suffers from sharing only part of the genome with each of the lines of the purebred selection candidates: 50% for two-way crossbreds and 25% for four-way crossbreds. Instead of two- or four-way crossbreds, pig breeding programs generally use three-way crossbreds. Since three-way crossbred animals share 50% of their genome with the sire line, results for the sire line of three-way crossbred programs will be equal to those for two-way crossbreds in our study [13]. Similarly, results for the dam line of three-way crossbred programs will be equal to those for the four-way crossbreds [13]. Thus, although we did not simulate three-way crossbreds, the results of this study can be easily extended to three-way crossbreds.

We simulated the traits based on only additive effects. However, allele substitution effects were simulated to be different in purebreds vs. crossbreds by using an ${r}_{pc}$ different from 1. Differences in allele substitution effects between purebred and crossbred populations can result from non-additive effects [7, 8] and differences in the environment in which the animals are raised [4–6]. The contribution of non-additive effects (dominance and epistasis) versus genotype-by-environment effects on ${r}_{pc}$ is not known. However, extensive and accurate estimates of ${r}_{pc}$ are available [10] and using this information, it enabled us to realistically model a pig population.

In this study, we used the same ${r}_{pc}$ for the different crossbred reference populations. The value of ${r}_{pc}$ due to dominance depends on the difference in allele frequencies between the purebred line of interest and the mated line [41, 42]. There is no reason why this difference would be larger when the mated line is crossbred instead of purebred. Therefore, with additive and dominance gene action, we do not expect a systematic difference in the value of ${r}_{pc}$ for two-way crossbreds compared to four-way crossbreds. However, the difference in allele frequencies between two purebred lines could become larger over time due to drift and selection, which reduces the value of ${r}_{pc}$. Although changes in allele frequencies are expected to be small over a limited number of generations [40, 43], dominance gene action can result in slight differences in ${r}_{pc}$ between crossbred populations generated from purebred ancestors of different generations, which was not taken into account in this study. Thus in principle, we assumed that only genotype-by-environment interactions contributed to ${r}_{pc}$.

We assumed that the line-origin of the alleles in the crossbreds could be derived without error. Vandenplas et al*.* [44] developed a method to assign line-origin to alleles based on haplotypes. For this method, the more distantly related are the parental lines, the more the haplotype frequencies differ between the lines, which makes it easier to track the line-origin of the haplotypes and their alleles in the crossbreds. The number of multiplication steps that occur in the purebred lines prior to crossing has no effect on the assignment of line-origin, because it only depends on the composition of the crossbred genome. In studies using real data, 95.2% of the alleles of three-way crossbreds pigs [45] and 91.1% of the alleles of three-way crossbred broilers [46] could be assigned to one of the lines using this method. The lower percentage in broilers was probably because the number of purebred genotypes available was small for one line [46]. Since the correct line origin is not known in real data, the accuracy of assigning the line-origin was investigated in a simulation study, which showed that the line-origin of an allele could be correctly assigned for 94.3 to 97.2% of the alleles in three-way crossbreds, with a higher percentage when the lines were more distantly related [44]. Collectively, these studies showed that it is possible to correctly assign line-origin for at least 95% of the alleles in the crossbred animals when at least 1000 animals are genotyped per purebred line. Incorrectly assigned alleles are, in the worst case, completely uninformative, but in general, genetic correlations between the parental lines are higher than 0, which means that even incorrectly assigned alleles can still contain some information. Therefore, we expect that when 95% of the alleles are correctly assigned, the accuracy of genomic prediction is still at least 95% of the accuracy with perfect assignment of alleles. Or, in other words, our assumption of being able to correctly assign the line-origin of alleles may have resulted in only a slight upward bias of accuracy for all crossbred scenarios.

### Bulmer equilibrium

Prior selection in the nucleus population was on purebred performance and a Bulmer equilibrium [40] was reached within about three generations. The resulting reduction in genetic variance in the nucleus population was larger for purebred performance than for crossbred performance because ${r}_{pc}$ was less than 1. Therefore, in generation 8, Bulmer-equilibrium genetic variances and heritabilities were larger for crossbred performance (${h}^{2}$ = 0.14 for ${r}_{pc}$ = 0.75, and ${h}^{2}$ = 0.16 for ${r}_{pc}$ = 0.5) than for purebred performance (${h}^{2}$ = 0.11). This was beneficial for the accuracy obtained with crossbred reference populations. However, if a crossbred reference population was used for multiple generations, the Bulmer equilibrium would become stronger for crossbred performance and, at equilibrium, the genetic variance and heritability for crossbred performance would be lower. This indicates that the slight benefit of a higher heritability for the crossbred populations will not persist over generations. We investigated the impact of this by predicting the accuracy of the different reference populations using the same heritability of 0.11 for all reference populations, instead of the different current Bulmer-equilibrium heritabilities (as done for Fig. 5b and d). This mainly affected the accuracies for small reference population sizes, for which the purebred reference population now slightly outperformed all crossbred reference populations when ${r}_{pc}$ was 0.75. For larger sizes of reference populations, the impact of differences in the Bulmer equilibrium heritability was small.

### Designing reference populations

Deciding on the best genotyping strategy is important when optimizing breeding programs [47]. In order to do so, the costs of each strategy must be considered. It is important to note that the same crossbred reference population can be used for multiple lines. For example, genotyping 2000 purebred animals for two separate within-line reference populations is just as expensive as genotyping 4000 two-way crossbred animals for a crossbred reference population that can be used for both lines. When using four-way crossbred animals, the number of genotyped crossbreds can be even four times larger. This means that the accuracy of 2000 purebred animals should be compared with that of 8000 four-way crossbred animals, which shows a clear benefit of the four-way crossbreds reference population, even when ${r}_{pc}$ is 0.75 (Fig. 5a). Thus, the benefit of using a crossbred reference population is likely larger when comparing scenarios that require the same total investment.

A crossbred reference population benefits from expressing the breeding goal trait [14, 25], but suffers from a lower genetic relatedness with the purebred selection candidates than a purebred reference population [16–18]. The balance between these two factors determines whether a crossbred or purebred reference population is beneficial for predicting breeding values for crossbred performance of purebred selection candidates. However, this balance depends on the size of the reference population, as shown by our results (Fig. 5), because an infinitely large crossbred reference population can theoretically result in a maximum accuracy of 1 [22, 23], compared to ${r}_{pc}$ for a purebred reference population [10]. With a large reference population, accuracy approaches its maximum and the benefit of recording the breeding goal trait outweighs the disadvantage of a lower genetic relatedness. With small reference population sizes, accuracy is strongly affected by the relatedness between the reference and selection populations, which is a disadvantage for the crossbred reference population. Thus, with smaller ${r}_{pc}$, the size at which the crossbred reference population outperforms the purebred reference population becomes smaller.

In practice, crossbred animals are raised in more variable environments, resulting in larger environmental variance components [11] and the environmental variance may even be heterogeneous across farms [48]. Therefore, heritability in commercial crossbred populations is often expected to be lower than in purebred populations [11, 49]. Our results show that, in this case, the benefit of a crossbred reference population occurred only when ${r}_{pc}$ was 0.5, but not 0.75 (Fig. 8). This shows that when heritability in crossbred populations is lower than in purebred populations, ${r}_{pc}$ has to be lower or the reference population larger in order to see a benefit of the crossbred reference population.

### Estimates of ${{\varvec{M}}}_{{\varvec{e}}}$ and predicted accuracy

The second aim of this study was to investigate the ability to rank different scenarios correctly by using deterministic equations to predict the accuracy of GEBV of purebred selection candidates for crossbred performance. For these predictions, the ${M}_{e}$ between the selection candidates and reference populations had to be estimated. Our results show that the ${M}_{e}$ between two-way crossbreds and purebreds was roughly double that for a purebred population with the same number of generations removed from the purebred selection candidates. This implies that variation in relationships between purebreds and two-way crossbreds is 50% of the variation in relationships among purebreds. This occurs because the variation in relationships among purebreds includes the variation contributed by both the sire and the dam, compared to the variation only contributed by the sire for a crossbred reference population. For the four-way crossbreds, the ${M}_{e}$ was four times as large as that for a purebred reference population because it only included variation contributed by the paternal grand sire. These results indicate that an estimate of the ${M}_{e}$ between purebred selection candidates and a crossbred reference population can be obtained based on the ${M}_{e}$ within a purebred line between generations. This means that only genotypes of purebred animals across multiple generations are required to obtain an estimate of the value of ${M}_{e}$ between purebreds and crossbreds. Based on this knowledge, the equation that we used to predict the accuracy became equivalent to the prediction equation derived by Vandenplas et al*.* [23], which uses the ${M}_{e}$ in purebreds times two to predict the accuracy for a two-way crossbred reference population.

The predicted accuracy of GEBV based on ${M}_{e}$ was always higher than the average empirical accuracy. One explanation for this is that the markers did not explain all the genetic variance in the selection candidates, which is assumed in the prediction formula that we used [21, 50]. This could be the case when markers and causal loci have different properties, such as allele frequency distributions [36]. However in our study, the difference in allele frequency distribution between markers and causal loci was limited. Another reason could be the high level of relatedness within the reference populations [51], which increases the overlap in information in the reference population. This suggests that the effective number of animals in the reference population may be smaller than the actual number of animals. Most important for our study is, however, that the ranking of the scenarios was similar for the empirical and predicted accuracy when the reference population was large. When the reference population was smaller, the accuracy of the purebred reference population was over-predicted to a larger extent than the accuracy of the crossbred reference population. This is probably due to the number of strong relationships between the reference and selection animals, which decreased fast when the size of the purebred reference population was reduced, but not when using a crossbred reference population. Specifically, all parents of the selection candidates were included in purebred reference populations of 2400 animals or more, but not in the smaller reference populations. Altogether, our results indicate that, although the accuracy is overestimated, the predicted accuracy can rank the different crossbred and purebred reference populations correctly when the reference population is large and can give some preliminary insights on the optimal reference population structure.

### Bias of estimated breeding values

Our results show that GEBV of purebred animals for crossbred performance are subject to considerable bias, especially for small reference population sizes and a high level of ${r}_{pc}$ (Table 2). This bias is probably a result of using base generation allele frequencies and variance components that accounted for the reduction in genetic variance over generations as a result of genome-wide changes in allele frequency (drift) since the base generation, but not for the Bulmer effect and also not for a greater change in allele frequencies at loci that affect the trait [40]. This is supported by the result that the bias was larger for a crossbred reference population when ${r}_{pc}$ was 0.75 than when ${r}_{pc}$ was 0.5, because the crossbred variance components were more affected by the Bulmer effect when ${r}_{pc}$ was higher. Moreover, the biases were lower when the genetic variance components were estimated and the reference population was large (see Additional file 5). Since only one generation with phenotypes and genotypes was included in the reference population, the data on which selection was based were not included in the model and, as a result, did not account for the Bulmer effect. When the reference population was larger, biases were generally lower. This is probably because the shrinkage of EBV becomes less dependent on variance components when the data contains more information.

## Conclusions

Altogether, we conclude that the benefit of a crossbred reference population over a purebred reference population for prediction of GEBV of purebred selection candidates for crossbred performance increases when the crossbred population is more related to the purebred selection candidates, when ${r}_{pc}$ is lower, and when the reference population is larger. When ${r}_{pc}$ is relatively low (≤ 0.5), a crossbred reference population is expected to be superior to a purebred reference population. When ${r}_{pc}$ is greater than 0.5, the benefit of a crossbred reference population compared to a purebred reference population depends on the size of the reference population. This shows that there is an interaction between size of the reference population size and the magnitude of ${r}_{pc}$ with respect to their impact on the accuracy of GEBV. Prediction equations based on ${M}_{e}$ provide preliminary insights on the ranking of different reference populations in terms of obtained accuracy, provided that reference populations are large. When comparing scenarios, it is also important to consider that a single crossbred reference population can be used for multiple purebred lines, while a purebred reference population can be used for only one line.

## Supplementary information

**Additional file 1.** Simulation of the TGV coming from the sire for four-way crossbreds. Explanation of how the TGV coming from the sire was simulated for the four-way crossbreds.

**Additional file 2.** Programs and seeds to simulate data. This file contains the input file used for QMSim, the Fortran-programs to simulate phenotypes and create new generations and the seeds for the different programs in each of the replicates

**Additional file 3.** Partial relationships. Histogram of the partial relationships for purebreds, two-way crossbreds and four-way crossbreds.

**Additional file 4.** Benefit of crossbred over purebred reference population. R-script to make a contour plot of the benefit of a crossbred over a purebred reference population as a function of ${r}_{pc}$ and reference population size.

**Additional file 5.** Results with estimated genetic variance components. Average accuracies and regression coefficients for purebreds, two-way-crossbreds or four-way crossbred reference population when all variance components were estimated.

## Data Availability

All scripts used to generate the data during this study are included in Additional file 2. This file contains the input file used for QMSim, the Fortran-programs to simulate phenotypes and create new generations and the seeds for the different programs in each of the replicates.

## References

[1] Smith C (1964). The use of specialised sire and dam lines in selection for meat production. Anim Sci.

[2] Dickerson GE. Inbreeding and heterosis in animals. In Proceedings of an Animal Breeding Symposium in Honor of Jay Lush: 29 July 1972; Blacksburg; 1973.

[3] Sellier P (1976). The basis of crossbreeding in pigs; a review. Livest Prod Sci.

[4] Dekkers JC (2007). Marker-assisted selection for commercial crossbred performance. J Anim Sci.

[5] Merks JWM (1989). Genotype × environment interactions in pig breeding programmes. VI. Genetic relations between performances in central test, on-farm test and commercial fattening. Livest Prod Sci..

[6] Rothschild MF, Ruvinsky A (2011). The genetics of the pig.

[7] Wei M, van der Werf JHJ, Brascamp EW (1991). Relationship between purebred and crossbred parameters: II Genetic correlation between purebred and cross bred performance under the model with two loci. J Anim Breed Genet.

[8] Fisher RA (1919). The correlation between relatives on the supposition of Mendelian inheritance. Trans Roy Soc Edinburgh.

[9] Fisher RA (1930). The genetical theory of natural selection.

[10] Wientjes YCJ, Calus MPL (2017). Board invited review: The purebred-crossbred correlation in pigs: A review of theory, estimates, and implications. J Anim Sci.

[11] Wei M, van der Werf JH (1995). Genetic correlation and heritabilities for purebred and crossbred performance in poultry egg production traits. J Anim Sci.

[12] Zumbach B, Misztal I, Tsuruta S, Holl J, Herring W, Long T (2007). Genetic correlations between two strains of Durocs and crossbreds from differing production environments for slaughter traits. J Anim Sci.

[13] Ibánẽz-Escriche N, Fernando RL, Toosi A, Dekkers JCM (2009). Genomic selection of purebreds for crossbred performance. Genet Sel Evol.

[14] van Grevenhof IE, van der Werf JH (2015). Design of reference populations for genomic selection in crossbreeding programs. Genet Sel Evol.

[15] Bijma P, Woolliams JA, Van Arendonk JAM (2001). Genetic gain of pure line selection and combined crossbred purebred selection with constrained inbreeding. Anim Sci.

[16] Pszczola MJ, Strabel T, Mulder HA, Calus MPL (2012). Reliability of direct genomic values for animals with different relationships within and to the reference population. J Dairy Sci.

[17] Wientjes YCJ, Veerkamp RF, Calus MPL (2013). The effect of linkage disequilibrium and family relationships on the reliability of genomic prediction. Genetics.

[18] Clark SA, Hickey JM, Daetwyler HD, van der Werf JHJ (2012). The importance of information on relatives for the prediction of genomic breeding values and the implications for the makeup of reference data sets in livestock breeding schemes. Genet Sel Evol.

[19] Wolc A (2014). Understanding genomic selection in poultry breeding. Worlds Poult Sci J.

[20] Daetwyler HD, Villanueva B, Woolliams JA (2008). Accuracy of predicting the genetic risk of disease using a genome-wide approach. PLoS One.

[21] Goddard ME (2009). Genomic selection: Prediction of accuracy and maximisation of long term response. Genetica.

[22] Wientjes YCJ, Veerkamp RF, Bijma P, Bovenhuis H, Schrooten C, Calus MPL (2015). Empirical and deterministic accuracies of across-population genomic prediction. Genet Sel Evol.

[23] Vandenplas J, Windig JJ, Calus MPL (2017). Prediction of the reliability of genomic breeding values for crossbred performance. Genet Sel Evol.

[24] Sevillano CA, Vandenplas J, Bastiaansen JWM, Bergsma R, Calus MPL (2017). Genomic evaluation for a three-way crossbreeding system considering breed-of-origin of alleles. Genet Sel Evol.

[25] Duenk P, Calus MPL, Wientjes YCJ, Breen VP, Henshall JM, Hawken R (2019). Validation of genomic predictions for body weight in broilers using crossbred information and considering breed-of-origin of alleles. Genet Sel Evol.

[26] Sargolzaei M, Schenkel FS (2009). QMSim: a large-scale genome simulator for livestock. Bioinformatics.

[27] Bosse M, Megens HJ, Madsen O, Paudel Y, Frantz LAF, Schook LB (2012). Regions of homozygosity in the porcine genome: Consequence of demography and the recombination landscape. PLoS Genet.

[28] Meuwissen THE, Hayes BJ, Goddard ME (2001). Prediction of total genetic value using genome-wide dense marker maps. Genetics.

[29] ten Napel J, Vandenplas J, Lidauer M, Stranden I, Taskinen M, Mäntysaari E, et al. MiXBLUP, user-friendly software for large genetic evaluation systems – Manual V2.1–2017–08. Wageningen; 2017.

[30] VanRaden PM (2008). Efficient methods to compute genomic predictions. J Dairy Sci.

[31] Groenen MAM, Megens HJ, Zare Y, Warren WC, Hillier LW, Crooijmans RPMA (2011). The development and characterization of a 60K SNP chip for chicken. BMC Genomics.

[32] Matukumalli LK, Lawley CT, Schnabel RD, Taylor JF, Allan MF, Heaton MP (2009). Development and characterization of a high density SNP genotyping assay for cattle. PLoS One.

[33] Ramos AM, Crooijmans RPMA, Affara NA, Amaral AJ, Archibald AL, Beever JE (2009). Design of a high density SNP genotyping assay in the pig using SNPs identified and characterized by next generation sequencing technology. PLoS One.

[34] Gilmour AR, Gogel BJ, Cullis BR, Welham SJ, Thompson R. ASReml user guide release 4.1. Hemel Hempstead: VSN International Ltd; 2015.

[35] Daetwyler HD, Pong-Wong R, Villanueva B, Woolliams JA (2010). The impact of genetic architecture on genome-wide evaluation methods. Genetics.

[36] Wientjes YCJ, Bijma P, Veerkamp RF, Calus MPL (2016). An equation to predict the accuracy of genomic values by combining data from multiple traits, populations, or environments. Genetics.

[37] Goddard ME, Hayes BJ, Meuwissen THE (2011). Using the genomic relationship matrix to predict the accuracy of genomic selection. J Anim Breed Genet.

[38] Badke YM, Bates RO, Ernst CW, Schwab C, Steibel JP (2012). Estimation of linkage disequilibrium in four US pig breeds. BMC Genomics.

[39] Veroneze R, Lopes PS, Guimarães SEF, Silva FF, Lopes MS, Harlizius B (2013). Linkage disequilibrium and haplotype block structure in six commercial pig lines. J Anim Sci.

[40] Bulmer MG (1971). The effect of selection on genetic variability. Am Nat.

[41] Christensen OF, Nielsen B, Su G, Xiang T, Madsen P, Ostersen T (2019). A bivariate genomic model with additive, dominance and inbreeding depression effects for sire line and three-way crossbred pigs. Genet Sel Evol.

[42] Duenk P. Genetics of crossbreeding. PhD thesis. Wageningen University and Research; 2020.

[43] Falconer DS, Mackay TFC (1996). Introduction to quantitative genetics.

[44] Vandenplas J, Calus MPL, Sevillano CA, Windig JJ, Bastiaansen JWM (2016). Assigning breed origin to alleles in crossbred animals. Genet Sel Evol.

[45] Sevillano CA, Vandenplas J, Bastiaansen JWM, Calus MPL (2016). Empirical determination of breed-of-origin of alleles in three-breed cross pigs. Genet Sel Evol.

[46] Calus MPL, Vandenplas J, Hulsegge I, Borg R, Henshall JM, Hawken R (2019). Assessment of sire contribution and breed-of-origin of alleles in a three-way crossbred broiler dataset. Poultry Sci.

[47] Knol EF, Nielsen B, Knap PW (2016). Genomic selection in commercial pig breeding. Anim Front.

[48] Márquez GC, Haresign W, Davies MH, Roehe R, Bünger L, Simm G (2015). Heterogeneous variances and genetics by environment interactions in genetic evaluation of crossbred lambs. Animal.

[49] Lourenco DAL, Tsuruta S, Fragomeni BO, Chen CY, Herring WO, Misztal I (2016). Crossbreed evaluations in single-step genomic best linear unbiased predictor using adjusted realized relationship matrices. J Anim Sci.

[50] Daetwyler HD. Genome-wide evaluation of populations. PhD thesis. Wageningen University; 2009.

[51] van den Berg I, Meuwissen THE, MacLeod IM, Goddard ME (2019). Predicting the effect of reference population on the accuracy of within, across, and multibreed genomic prediction. J Dairy Sci.

