# Supplementary material for: Optimizing genomic reference populations to improve crossbred performance
Source: Genet Sel Evol. 2020 Nov 6;52:65. doi: 10.1186/s12711-020-00573-3 (PMC7648379; doi:10.1186/s12711-020-00573-3)
Supplement: Supplementary file 1 — Additional file 1. Simulation of the TGV coming from the sire for four-way crossbreds. Explanation of how the TGV coming from the sire was simulated for the four-way crossbreds. [file 12711_2020_573_MOESM1_ESM.docx]

# Additional file 1

## Simulation of the TGV coming from the sire for four-way crossbreds

Four-way crossbred animals on average receive 25% of their genome from each of their grandparents. Although exactly 50% of the genome originates from each of the parents, there is sampling of the proportion obtained from each of the grandparents because of segregation in the F1-parents. In our simulations, only the part of the genome originating from the paternal grand sire (PGS) was simulated with genotypes, while the rest was simulated as for a polygenic trait. Thus, for on average 50% of the paternal haplotype the alleles originated from the PGS and were known. We multiplied those allele counts with the corresponding allele substitution effects to calculate the TGV coming from the PGS (TGV_PGS_).

The other ~50% of the paternal haplotype originated from the PGD. This mean that there is variation in the proportion of the paternal haplotype originating from the PGD, and in the overlap of that proportion between sibs. This proportion has to be taken into account when sampling the polygenic TGV coming from the PGD (TGV_PGD_). The expected value of TGV_PGD_ is proportional to the part of the genome that originated from the PGD. The resemblance between the TGV_PGD_ of sibs is proportional to the overlap in the part of the genome originating from the PGD between sibs.

Therefore, we sampled the TGV_PGD_ for all sibs together from a multivariate normal distribution. First, for each four-way crossbred animal *i*, we calculated the part of the paternal haplotype that originated from the PGD (*p_PGD,i_*), and the overlap of this part between sibs (*p_PGD,i-j_*). Next, the TGV_PGD_ were sampled simultaneously for all 30 sibs (full sibs and paternal half-sibs), using

$$\boldsymbol{TG}\boldsymbol{V}_{PGD}\boldsymbol{\sim}MVN\left( \boldsymbol{\mu,}\boldsymbol{\sigma}^{2} \right)\boldsymbol{,}$$

where *µ_i_* is *p_PGD,i_**TGV_sire,PGD_, diagonal elements of ***σ^2^*** are $\left[ p_{PGD,i}-\left( p_{PGD,i} \right)^{2} \right]*\frac{1}{2}\sigma_{A_{CB}}^{2}$, off-diagonal elements of ***σ^2^*** are $\left[ p_{PGD,i-j}-\left( p_{PGD,i}*p_{PGD,j} \right) \right]*\frac{1}{2}\sigma_{A_{CB}}^{2}$, and TGV_sire,PGD_ represents the TGV of the sire coming from the PGD. Note that this term was simulated in the generation before to create the two-way crossbred animals. In the expression for ***σ^2^***, the term $\left( p_{PGD,i}*p_{PGD,j} \right)$ represents a correction for the expected genome sharing between sibs, which is already accounted for in ***μ***. Finally, TGV_Sire_  = TGV­_PGS_ + TGV_PGD_.
