# Supplementary material for: Optimizing genomic reference populations to improve crossbred performance
Source: Genet Sel Evol. 2020 Nov 6;52:65. doi: 10.1186/s12711-020-00573-3 (PMC7648379; doi:10.1186/s12711-020-00573-3)
Supplement: Supplementary file 3 — Additional file 3. Partial relationships. Histogram of the partial relationships for purebreds, two-way crossbreds and four-way crossbreds. [file 12711_2020_573_MOESM3_ESM.docx]

# Additional file 3


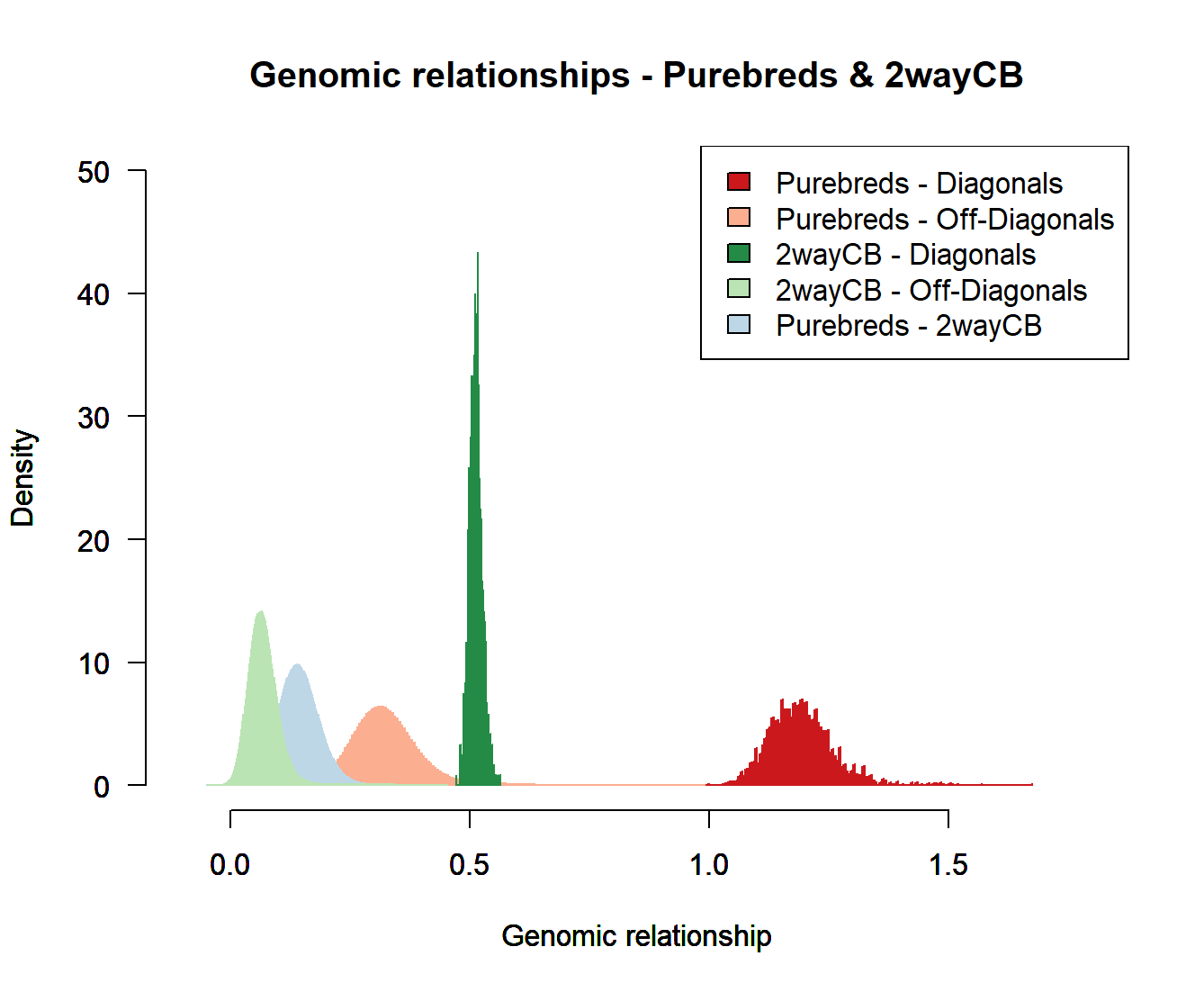


**Figure S3.1 – Histogram of relationship in partial relationship matrix with two-way crossbreds.**


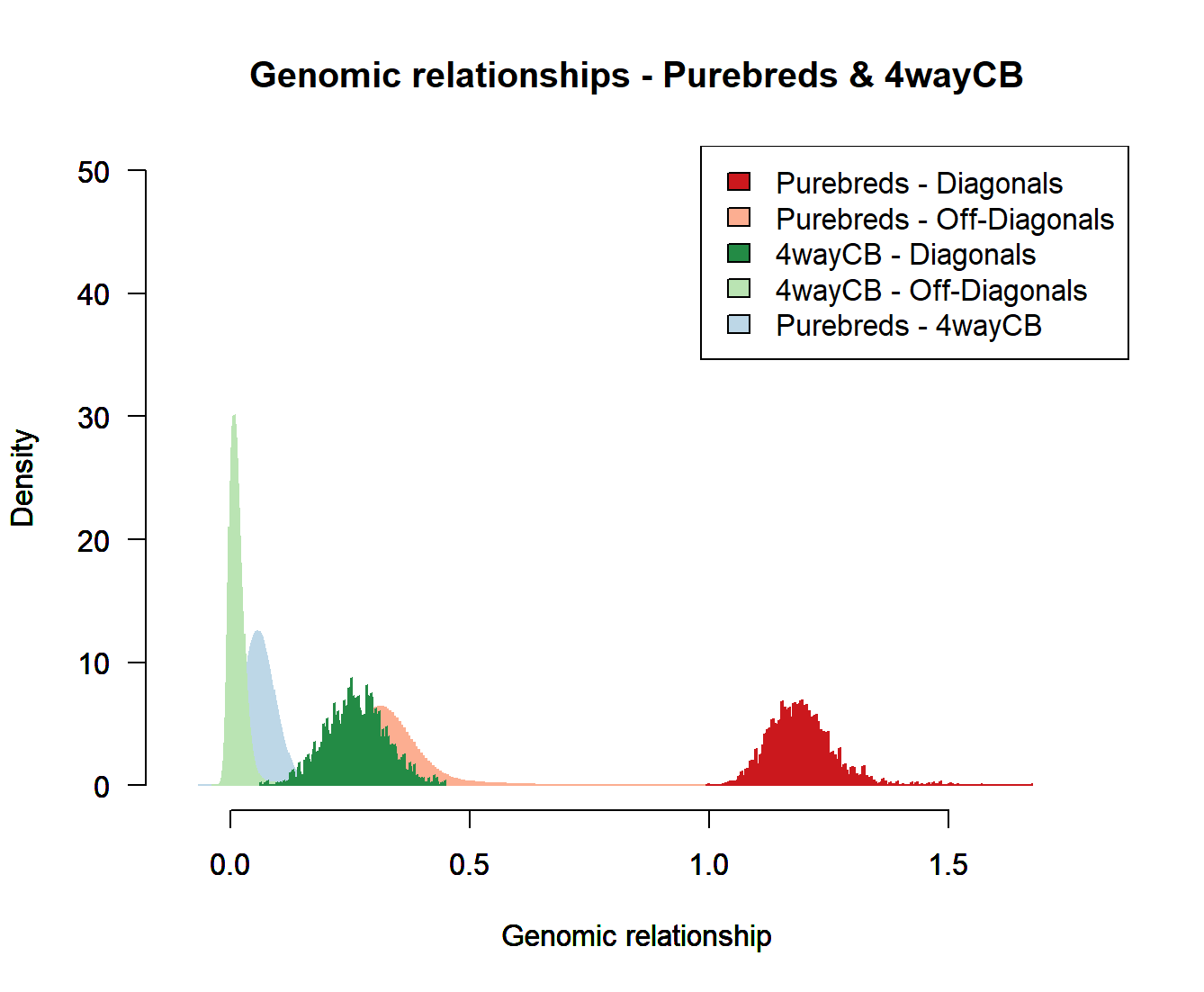


**Figure S3.2 – Histogram of relationship in partial relationship matrix with four-way crossbreds.**
