# Supplementary material for: Optimizing genomic reference populations to improve crossbred performance
Source: Genet Sel Evol. 2020 Nov 6;52:65. doi: 10.1186/s12711-020-00573-3 (PMC7648379; doi:10.1186/s12711-020-00573-3)
Supplement: Supplementary file 5 — Additional file 5. Results with estimated genetic variance components. Average accuracies and regression coefficients for purebreds, two-way-crossbreds or four-way crossbred reference population when all variance components were estimated. [file 12711_2020_573_MOESM5_ESM.docx]

# Additional file 5


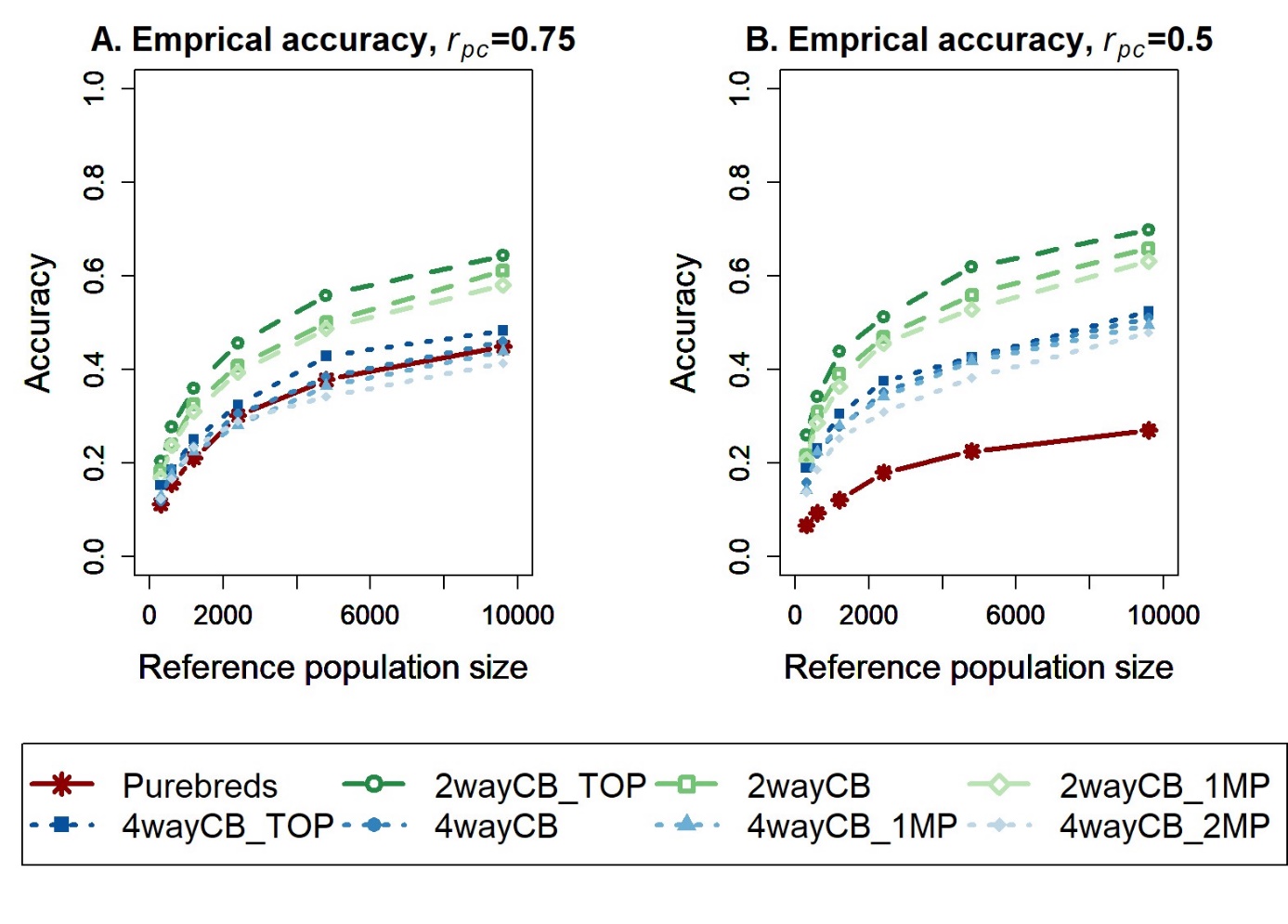


**Figure S5.1** - Average empirical accuracy of estimated breeding values of purebred selection candidates for crossbred performance when variance components are estimated. The reference population consisted of purebreds (PB), two-way crossbreds (CB) derived from the same sires as used in the nucleus (2wayCB_TOP), different sires with zero (2wayCB) or one multiplication step (2wayCB_1MP), four-way crossbreds derived from the same sires as used in the nucleus population (4wayCB_TOP), different sires with zero (4wayCB), one (4wayCB_1MP), or two multiplication steps (4wayCB_2MP). The purebred-crossbred correlation was equal to 0.75 (a and b) or 0.5 (c and d). Heritability was 0.2 in the purebred and crossbred populations. Averages were calculated across 50 replicates.

**Table S5.1 –** Regression coefficients of true breeding values on genomic estimated breeding values of purebred selection candidates for crossbred performance for different reference populations when estimating the genetic variance.

| \| **Scenario** \| **Reference population size** \| \| \| \| \| \| \| --- \| --- \| --- \| --- \| --- \| --- \| --- \| \|  \| **9600** \| **4800** \| **2400** \| **1200** \| **600** \| **300** \| \| \| **Purebred-crossbred correlation is 0.75** \| \| \| \| \| \| \| \| **Purebred** \| 0.69 \| 0.68 \| 0.71 \| 0.74^a^ \| 31131.74^a^ \| 50459.93^a^ \| \| \| **2wayCB_TOP** \| 0.94 \| 0.91 \| 1.00 \| 48751.54^a^ \| 161103.88^a^ \| 164140.17^a^ \| \| \| **2wayCB** \| 0.94 \| 0.95 \| 1.01 \| 1.95^a^ \| 145786.05^a^ \| 149453.44^a^ \| \| \| **2wayCB_1MP** \| 0.91 \| 0.93 \| 0.85 \| 0.86 \| 1.23^a^ \| 110726.22^a^ \| \| \| **4wayCB_TOP** \| 0.82 \| 0.86 \| 0.94 \| 72863.84^a^ \| 142015.28^a^ \| 136967.75^a^ \| \| \| **4wayCB** \| 0.82 \| 0.89 \| 30.21^a^ \| 80210.98^a^ \| 223152.21^a^ \| 53959.05^a^ \| \| \| **4wayCB_1MP** \| 0.80 \| 0.82 \| 0.86 \| 3000.34^a^ \| 135969.99^a^ \| 170576.69^a^ \| \| \| **4wayCB_2MP** \| 0.69 \| 0.71 \| 0.83 \| 19183.35^a^ \| 30155.12^a^ \| 60925.01^a^ \| \| \|  \|  \|  \|  \|  \|  \|  \| \| \| **Purebred-crossbred correlation is 0.5** \| \| \| \| \| \| \| \| **Purebred** \| 0.45 \| 0.44 \| 0.46 \| 0.45 \| 20229.77^a^ \| 20554.20^a^ \| \| \| **2wayCB_TOP** \| 0.95 \| 0.98 \| 0.96 \| 1.16 \| 50899.52^a^ \| 102751.4 ^a^ \| \| \| **2wayCB** \| 0.96 \| 0.98 \| 0.99 \| 1.04 \| 83185.96^a^ \| 208588.85^a^ \| \| \| **2wayCB_1MP** \| 0.98 \| 0.93 \| 0.94 \| 0.93 \| 48160.37^a^ \| 228549.01^a^ \| \| \| **4wayCB_TOP** \| 0.92 \| 1.04 \| 1.00 \| 22188.31^a^ \| 93261.65^a^ \| 210889.71^a^ \| \| \| **4wayCB** \| 0.89 \| 0.87 \| 0.96 \| 63186.09^a^ \| 109302.09^a^ \| 141907.55^a^ \| \| \| **4wayCB_1MP** \| 0.87 \| 0.89 \| 0.98 \| 78293.60^a^ \| 139804.08^a^ \| 113721.93^a^ \| \| \| **4wayCB_2MP** \| 0.87 \| 0.80 \| 0.98 \| 20070.81^a^ \| 99651.22^a^ \| 201197.26^a^ \| \| |
| --- | --- | --- | --- | --- | --- | --- | --- | --- | --- | --- | --- | --- | --- | --- | --- | --- | --- | --- | --- | --- | --- | --- | --- | --- | --- | --- | --- | --- | --- | --- | --- | --- | --- | --- | --- | --- | --- | --- | --- | --- | --- | --- | --- | --- | --- | --- | --- | --- | --- | --- | --- | --- | --- | --- | --- | --- | --- | --- | --- | --- | --- | --- | --- | --- | --- | --- | --- | --- | --- | --- | --- | --- | --- | --- | --- | --- | --- | --- | --- | --- | --- | --- | --- | --- | --- | --- | --- | --- | --- | --- | --- | --- | --- | --- | --- | --- | --- | --- | --- | --- | --- | --- | --- | --- | --- | --- | --- | --- | --- | --- | --- | --- | --- | --- | --- | --- | --- | --- | --- | --- | --- | --- | --- | --- | --- | --- | --- | --- | --- | --- | --- | --- | --- | --- | --- | --- | --- | --- | --- | --- | --- | --- | --- | --- | --- | --- | --- | --- | --- | --- | --- | --- | --- | --- | --- | --- | --- | --- | --- | --- | --- | --- | --- | --- | --- |

^a^ _­_In some replicates, the regression coefficient was large (>10), because the estimated genetic variance was converging to zero. This is caused by the low number of animals in the analyses, which made it difficult to estimate the genetic variance.
